# Comprehensive Functional Analysis of *Mycobacterium tuberculosis* Toxin-Antitoxin Systems: Implications for Pathogenesis, Stress Responses, and Evolution

**DOI:** 10.1371/journal.pgen.1000767

**Published:** 2009-12-11

**Authors:** Holly R. Ramage, Lynn E. Connolly, Jeffery S. Cox

**Affiliations:** 1 Department of Microbiology and Immunology, University of California San Francisco, San Francisco, California, United States of America; 2 Program in Microbial Pathogenesis and Host Defense, University of California San Francisco, San Francisco, California, United States of America; 3 Department of Medicine, Division of Infectious Diseases, University of California San Francisco, San Francisco, California, United States of America; Baylor College of Medicine, United States of America

## Abstract

Toxin-antitoxin (TA) systems, stress-responsive genetic elements ubiquitous in microbial genomes, are unusually abundant in the major human pathogen *Mycobacterium tuberculosis*. Why *M. tuberculosis* has so many TA systems and what role they play in the unique biology of the pathogen is unknown. To address these questions, we have taken a comprehensive approach to identify and functionally characterize all the TA systems encoded in the *M. tuberculosis* genome. Here we show that 88 putative TA system candidates are present in *M. tuberculosis*, considerably more than previously thought. Comparative genomic analysis revealed that the vast majority of these systems are conserved in the *M. tuberculosis* complex (MTBC), but largely absent from other mycobacteria, including close relatives of *M. tuberculosis*. We found that many of the *M. tuberculosis* TA systems are located within discernable genomic islands and were thus likely acquired recently via horizontal gene transfer. We discovered a novel TA system located in the core genome that is conserved across the genus, suggesting that it may fulfill a role common to all mycobacteria. By expressing each of the putative TA systems in *M. smegmatis*, we demonstrate that 30 encode a functional toxin and its cognate antitoxin. We show that the toxins of the largest family of TA systems, VapBC, act by inhibiting translation via mRNA cleavage. Expression profiling demonstrated that four systems are specifically activated during stresses likely encountered *in vivo*, including hypoxia and phagocytosis by macrophages. The expansion and maintenance of TA genes in the MTBC, coupled with the finding that a subset is transcriptionally activated by stress, suggests that TA systems are important for *M. tuberculosis* pathogenesis.

## Author Summary

Tuberculosis (TB) continues to be a major global health problem, causing 2 million deaths every year. A hallmark of TB pathogenesis is that the bacilli can enter into a slow or non-growing state in response to the host immune system. Because these persistent bacteria are resistant to antibiotic treatment, efforts to eliminate TB from the human population must include therapies to target dormant organisms as they can eventually resume replication to cause active disease. How *Mycobacterium tuberculosis*, the causative agent of TB, alters its replication dynamics in response to host cues is not understood. Toxin-antitoxin (TA) systems, which may control persistence in other bacteria, are massively expanded in *M. tuberculosis*, suggesting that they are important for TB pathogenesis. Surprisingly, the vast majority of these numerous TA systems are conserved only in pathogenic mycobacteria, suggesting their acquisition was important in *M. tuberculosis* evolution. Of the 88 putative TA systems identified, we show that 30 are functional in mycobacteria. A subset of these systems is activated upon exposure to stresses encountered during infection, indicating that specific TA systems are involved in adapting to environmental cues in the host. These genes are promising candidates for the development of novel therapies to target persistent bacteria.

## Introduction

Toxin-antitoxin (TA) systems are ubiquitous in prokaryotic genomes and have been proposed to play a role in several important cellular functions [1]. These systems typically consist of a two-gene operon encoding a toxic protein that targets an essential cellular function and an antitoxin that binds to and inhibits the toxin. Regulation of toxin activity is achieved through differential stability of the stable toxin and the unstable antitoxin [2]. In most cases, the antitoxin also acts as a transcriptional autorepressor of the operon, such that degradation of the antitoxin results in transcriptional induction of the TA genes. Most of what we know about TA systems has come from the pioneering work in *E. coli*, though their role in bacterial physiology is still controversial. Some of the genome-encoded systems are activated in response to environmental stress, resulting in cell stasis from which these cells can recover under more favorable growth conditions [2],[3]. In contrast, it has also been reported that the MazEF TA system participates in programmed cell death [3]–[6]. Importantly, the HipBA TA system has been implicated in the formation of persister cells, a subpopulation of bacteria that exhibit antibiotic tolerance in an otherwise susceptible population [7],[8] and may thus contribute to the long treatment times required to cure some infections. Because TA systems were discovered as plasmid stability elements, it has also been proposed that genomic TA loci may similarly stabilize or help to ensure the maintenance of genes encoded nearby in the genome [1],[9],[10]. Finally, it has also been postulated that these systems are simply selfish genetic elements that function only to maintain their own existence in a genome [1],[11]. Although these studies have provided a wealth of information regarding the function of TA systems in *E. coli*, their role in the physiology of other microbes remains largely unexplored.

The most common mechanism of TA system toxicity is mediated through mRNA cleavage, resulting in translation inhibition [2],[12],[13]. Two well-characterized TA system families of *E. coli*, MazEF and RelBE, have been shown to act via this mechanism and cleave specific three-nucleotide sequences [14],[15]. The toxins of the largest family of TA systems in *M. tuberculosis*, VapBC, contain PIN domains, a motif thought to be associated with ribonuclease function [16] and have been shown to block translation via mRNA cleavage [17]–[19]. Transient activation of these mRNases may allow the bacteria to adapt to stress not only by inhibiting replication, but also by degrading existing transcripts, allowing a rapid change in the metabolic program of the bacteria.

Given that a TA system is required for the formation of persisters in *E. coli*, it has been speculated that the TA systems of *M. tuberculosis* may govern cell division decisions during infection [10]. In the majority of individuals infected with *M. tuberculosis*, the bacteria initially grow and then establish a latent, asymptomatic infection that can persist for decades with the potential to reactivate later in life [20],[21]. These persistent bacteria are thought to adopt a slowly or non-replicating state in response to environmental stresses encountered in the host [22],[23], yet the mechanisms by which this non-replicating state is achieved are unknown. Because the majority of current antimicrobials require bacterial growth to exert their killing action, these non-replicating persistent bacteria are thought to comprise an important subpopulation of bacteria that is refractory to antibiotic therapy [24]. A similar antibiotic-tolerant state is elicited by TA system activation in other bacteria [25], suggesting that TA systems may contribute to the long duration of antibiotic therapy required to cure tuberculosis.

The most extensively studied culture condition to induce cell stasis in *M. tuberculosis* is hypoxia [26]–[28]. Gradual oxygen depletion in culture results in significant changes in metabolism and gene expression, leading to a non-replicative persistent (NRP) state [26]. Because bacteria experience an effectively hypoxic environment *in vivo* as a result of reduced oxygen availability and exposure to nitric oxide (NO), it is thought that hypoxia-induced NRP is similar to the *in vivo* state [21],[26],[29],[30]. Additionally, these two conditions result in a significant overlap in gene expression as they both induce the dormancy regulon, a set of genes under the control of the transcription factor DosR [26],[31]. Bacilli grown under hypoxia exhibit a tolerance to antimicrobial therapy [26]. Given that hypoxia results in a state of cell stasis and the formation of antibiotic-tolerant persisters, TA systems are prime candidates for mediating this transition both *in vitro* and *in vivo*.

Recent bioinformatics studies revealed that the *M. tuberculosis* genome encodes numerous TA system homologs and many PIN domain-containing proteins, far more than any other intracellular pathogen [16], [32]–[34]. Although these studies suggested that there has been a significant expansion of TA systems in *M. tuberculosis*, these analyses may have missed distantly related homologs and novel families of TA systems and thus the total number of TA systems in *M. tuberculosis* may be even greater. Additionally, how the *M. tuberculosis* genome evolved to acquire and maintain these TA systems during its evolution is unclear. To date, there has not been a comprehensive comparative analysis to determine whether the *M. tuberculosis* TA systems have been selectively maintained in the pathogens of this genus. TA systems are often associated with mobile genetic elements and are thus commonly acquired by horizontal gene transfer [16],[32], yet only three of the *M. tuberculosis* TA systems have been definitively assigned to a known genomic island [35],[36]. Although the evolutionary history of these genes is uncertain, the vast number of TA systems in *M. tuberculosis* evokes the question of whether the expansion of TA systems in *M. tuberculosis* plays an important role in the physiology of the bacteria.

A subset of the putative *M. tuberculosis* TA genes have been partially characterized but our knowledge of the full complement of TA systems thus far is very fragmented [12], [13], [37]–[39]. Therefore, a comprehensive and systematic analysis is needed to provide a foundation on which to investigate the role of this interesting gene family in *M. tuberculosis* biology. Although recent bioinformatic analyses have expanded the number of putative TA systems encoded in the *M. tuberculosis* genome [16],[32],[33], the key questions of how many of these genes encode functional TA systems and which of these systems are important in *M. tuberculosis* biology have not been addressed.

Here we report the results of a comprehensive strategy to identify and examine the putative TA systems encoded in the *M. tuberculosis* genome. Our approach revealed many more putative TA loci than previously appreciated. Expression of each of these systems in *M. smegmatis*, a fast-growing relative of *M. tuberculosis*, revealed a subset that encodes *bona fide* TA systems. Importantly, we identified three novel systems that were not previously recognized and bear no similarity to known TA genes, and thus may represent new families of TA systems. Additionally, by performing comparative genomic analysis across the mycobacterial genus, we made the striking discovery that the vast majority of these systems are conserved only in the MTBC and are absent from mycobacteria outside this complex, including closely-related pathogenic species. The acquisition and expansion of TA systems likely occurred coincident with or after speciation of the MTBC from the last common ancestor, suggesting an important role for these genes in *M. tuberculosis* evolution. We demonstrate that toxins with homology to RNases inhibit translation and have RNase activity *in vitro*, while a novel toxin likely functions via a different mechanism. Finally, we show that subsets of these genes are upregulated during hypoxia or macrophage infection, providing strong evidence that these systems are activated during specific stresses likely encountered in the host.

## Results

### The genome of *Mycobacterium tuberculosis* encodes several TA system homologs, as well as novel TA systems

To broadly search the *M. tuberculosis* genome for putative TA systems, we reasoned that a combination of approaches would be more powerful than a single strategy. To this end, we utilized three complementary approaches that took advantage of different characteristics of known TA systems. First, we performed PSI-BLAST searches of the *M. tuberculosis* genome using the toxin and antitoxin protein sequences from each of eight major TA system families: CcdBA, HigBA, HipBA, MazEF, ParDE, RelBE, VapBC, and Doc/PhD (Table S1). When possible, we used sequences from both distantly-related organisms (Gram-negative) and more closely-related organisms (Gram-positive, high-GC) to search for homologs. Second, we expanded this list to include PIN domain-containing proteins and toxin-antitoxin systems identified in previous analyses [16]. Finally, we used a sequence-independent approach to identify pairs of adjacent genes encoded in the *M. tuberculosis* genome that bear no homology to known TA systems but share a similar genomic organization [40]. This method, similar to an approach used to identify novel TA systems in *E. coli*, included constraints on size, orientation, and spacing of putative TA pairs. Candidate genes from all three methods were filtered using four criteria for the genomic organization of TA systems: 1) the putative toxin and antitoxin genes were adjacent to one another, 2) the two putative genes were separated by fewer than 150 bp, likely comprising an operon, 3) neither gene encoded a protein larger than 150 amino acids, and 4) the upstream gene (putative antitoxin) was smaller than the downstream gene (putative toxin). The last criterion was disregarded for cases in which there were small differences in size provided that both the putative toxin and antitoxin had conserved protein domains associated with their proposed function. Additionally, we made an exception in the case of the lone HigBA homolog, as the orientation of the toxin and antitoxin are reversed in this system [32].

**Figure 1:**
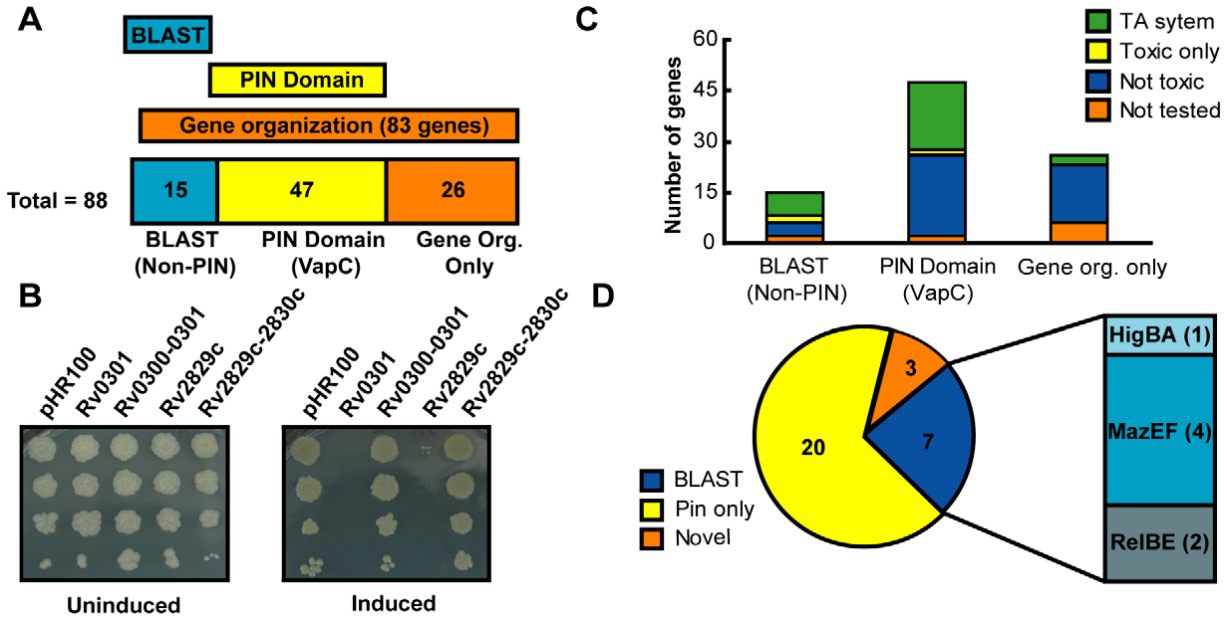
Identification and testing of putative TA systems. (A) Three approaches were used to identify putative TA systems. These are indicated as BLAST (Non-PIN; homologs found through BLAST analysis that do not contain PIN domains), PIN Domain (PIN domain-containing proteins), and Genome Org. Only (novel and not homologous to known TA systems). (B) *M. smegmatis* cultures with putative toxins or putative toxin-antitoxin pairs under the control of the inducible acetamidase promoter were serially diluted and plated on solid media with (right panel) or without (left panel) 0.2% acetamide. (C) Summary of toxin and antitoxin testing results: not tested (unable to PCR or clone gene products), not toxic (no toxin activity was detected), toxic only (toxicity was not relieved by the putative antitoxin), or a TA system (toxic activity relieved by antitoxin). (D) Functional TA systems were identified as novel, PIN domain-containing proteins, or homologs found by BLAST (non-PIN domain-containing proteins). Genes found by BLAST were further subdivided by the TA system family to which they belong.

**Figure 2:**
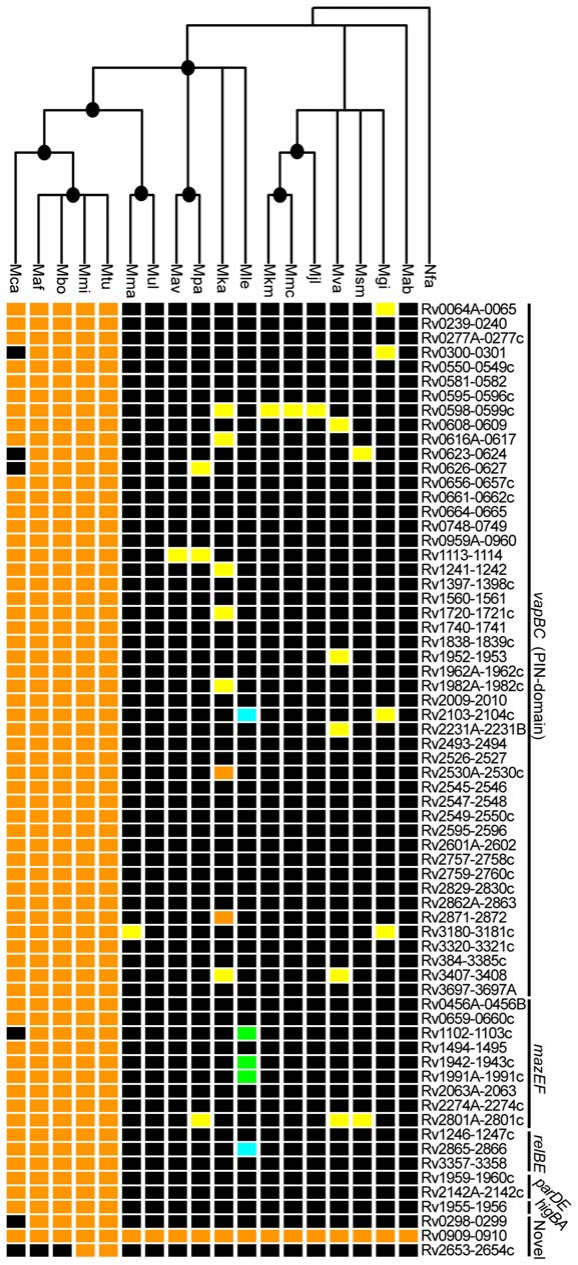
TA system conservation across the genus *Mycobacterium*. Phylogenetic tree based on 16S rDNA sequences showing conservation of TA systems. The tree was constructed using Neighbor-joining inference method and nodes supported by bootstrap values>70% (1,000 replicates) are shown. *Nocardia farcinica* (Nfa) was used as the outgroup. TA systems are arranged according to family (*vapBC*, *mazEF*, *relBE*, *parDE*, *higBA*, and novel); for details see Table S6. Orange represents orthologs (BLAST best reciprocal hits displaying synteny), yellow: BLAST best reciprocal hits residing in different genomic contexts (homologs), blue: pseudogenes residing in similar genomic context, green: pseudogenes residing in different genomic contexts, and black indicates no hits were detected by BLAST. Abbreviations: Nfa *Nocardia farcinica*; Mab *Mycobacterium abscessus*; Mgi *Mycobacterium gilvum*; Msm *Mycobacterium smegmatis*; Mva *Mycobacterium vanbaalenii*; Mjl *Mycobacterium* sp. JLS; Mmc *Mycobacterium* sp. MCS; Mkm *Mycobacterium* sp. KMS; Mle *Mycobacterium leprae*; Mka *Mycobacterium kansasii*; Mpa *Mycobacterium avium* str. k10; Mav *Mycobacterium avium* 104; Mul *Mycobacterium ulcerans*; Mma *Mycobacterium marinum*; Mtu *Mycobacterium tuberculosis*; Mmi *Mycobacterium microti*; Mbo *Mycobacterium bovis*; Maf *M. africanum*; Mca *Mycobacterium canetti*.

In total, we generated a list of 88 putative TA systems in *M. tuberculosis* (Figure 1A and Table S1). Our list includes 62 gene pairs that were identified by homology. In cases for which the putative toxins and antitoxins belonged to different TA families, the TA system homology was assigned based on the homology of the toxin gene (Table S1). Toxins that contain PIN domains are most closely related to the VapBC family and thus we have classified TA systems with toxins containing these domains as part of the VapBC family (Figure 2, Table S2, and Table S3). We identified an additional 26 putative systems that share no sequence similarity to known TA genes and thus may represent novel systems (Figure 1A and Table S2). Two other groups have incorporated homology-independent methods in addition to traditional homology-dependent searches to aid in the comprehensive identification of TA systems in prokaryotic genomes [33],[41]. Similar to the methods used here, Sevin and Barloy-Hubler utilized a sequence-independent approach that relies on the characteristic size, gene organization and spacing of known TA systems to develop an automated, web-based tool termed RASTA-Bacteria. Makarova, *et al.* used a novel approach that identifies pairs of genes that are significantly non-uniformly distributed in prokaryotic genomes. Comparison of our results with those of the above studies revealed that our methods identified a large number (23–28) of putative new TA systems, largely of the novel class, not identified in either the Makarova study nor deemed significant by RASTA-Bacteria (Figure S1).

### TA system expansion is unique to the species of the MTBC

To better define when in evolutionary history TA module expansion occurred, we examined both published and unpublished genome sequences spanning the genus *Mycobacterium* for orthologs and homologs of the known and newly identified TA systems present in *M. tuberculosis*. We included the draft genomes of several members of the MTBC, including *M. canetti*, which is the deepest diverging lineage of this group [42], as well as the genomes of *M. marinum* and *M. kansasii*, which are the most closely related mycobacterial species that lie outside the MTBC [35],[43]. Finally, completed genomes of rapidly growing environmental mycobacteria and the slow growing pathogens *M. leprae*, *M. ulcerans and M. avium* were included. We used reciprocal best BLAST hit and genomic context analyses [44] to identify orthologs and homologs of the toxin portion of 65 of the 84 putative *M. tuberculosis* TA systems described above. For all top BLAST hits, we then determined whether an associated antitoxin was encoded nearby in the genome. 23 putative toxin genes identified by genomic organization were excluded from this analysis as we found no supporting evidence based on either homology or over-expression experiments (see below) that they encoded functional toxins.

Analysis of the MTBC identified orthologs of nearly every TA system in each of the genomes analyzed (Figure 2 and Table S3). All of the TA systems were conserved in *M. microti*, and only one toxin, Rv2653c, which is encoded on a prophage, was absent in *M. bovis* and *M. africanum*. Six toxin genes appeared to be absent in *M. canetti*, including the prophage-encoded toxin Rv2653c. Analysis of the surrounding genomic sequences, however, revealed that sequences homologous to three of these genes, *Rv0624*, *Rv0627* and *Rv1102c*, were present in the *M. canetti* genome but had been disrupted by genomic rearrangements, including the insertion of transposon-like sequences (data not shown). The last two toxins absent from the *M. canetti* genome, Rv0299 and Rv0301, are encoded on a genomic island in *M. tuberculosis* that has previously been shown to be absent in several *M. canetti* strains (Table 1). These results suggest that the vast majority of TA systems were present in the progenitor of the MTBC. The finding that a small number of TA genes have been lost in *M. canetti* is consistent with its assignment as the deepest branching member of the MTBC lineage [42].

**Table 1:** Inferred genomic islands encoding TA systems and associated genes.

Genomic Island	TA systems encoded	Associated genes implicated in virulence or stress adaptation
*Rv0057-Rv0080* a	*Rv0064A-0065 (vapBC1)*	None
*Rv0298-Rv0303* b	*Rv0298-0299*	None
	*Rv0300-0301 (vapBC2)*	
*Rv0595c-Rv0614* b	*Rv0595c-0596c (vapBC4)*	None
	*Rv0598c-0599c (vapBC27)*	
	*Rv0608-0609 (vapBC28)*	
*Rv0656c-Rv0666* b	*Rv0656c-0657c (vapBC6)*	*Rv0666* c
	*Rv0659c-0660c (mazEF2)*	
	*Rv0661c-0662c (vapBC7)*	
	*Rv0664-0665 (vapBC8)*	
*Rv0739-Rv0750* b	*Rv0748-0749 (vapBC31)*	None
*Rv1397c-Rv1398c* b	*Rv1397c-1398c (vapBC10)*	None
*Rv1942-Rv2028* a	*Rv1942c-1943c (mazEF5)*	*mce3* operon^d^
*(Rv1988-Rv1991c)* b	*Rv1955-1956 (higBA)*	*otsB1* e
	*Rv1959c-1960c (parDE1)*	*dosT* and dormancy regulon member *fdxA* f
	*Rv1962A-1962c (vapBC35)*	
	*Rv1982A-Rv1982c (vapBC36)*	
	*Rv1991A-1991c (mazEF6)*	
	*Rv2009-2010 (vapBC15)*	
*Rv2100-2108* a	*Rv2103c-2104c (vapBC37)*	
*Rv2491-Rv2494* b	*Rv2493-2494 (vapBC38)*	
*Rv2801c-Rv2824c* b	*Rv2801c-2802c (mazEF9)*	Direct repeat (DR) locus; phage resistance^g^
*(Rv2802-2830)* a		*Rv2808* c, *Rv2813* c
*Rv2871-Rv2872* b	*Rv2871-2872 (vapBC43)*	
*Rv3320c-Rv3324c* a	*Rv3320c-3321c (vapBC44)*	Molybdopterin locus II^h^

**a** [35].

**b** [46].

**c** [57].

**d** [61].

**e** [62].

**f** [63].

**g** [64].

**h** [65].

TA systems encoded in previously described genomic islands. Genomic islands identified by parametric or combined parametric and phylogenetic methods [35],[46] were examined for the presence of the TA systems identified in this work.

BLASTP analysis identified putative toxins in several mycobacterial genomes outside of the MTBC (Figure 2 and Table S3). In most cases, however, the surrounding genomic regions did not demonstrate conservation of gene content and order with the *M. tuberculosis* genome, suggesting that these related TA systems were acquired independently in each bacterial species rather than having evolved from a common ancestral acquisition event. Alternatively, because TA systems are often associated with mobile genetic elements, these systems may have been present in a common ancestor and subsequently moved in the genome as each species diverged. Three of the five TA systems (coding and pseudogenes) identified in *M. leprae* and two of the 12 TA systems found in the *M. kansasii* genome are in regions with similar gene content and order to that of *M. tuberculosis*, arguing that they evolved from a common ancestral acquisition event. Intriguingly, the only TA module encoded in all genomes analyzed was the novel TA system Rv0909-Rv0910, identified in the homology-independent search.

Strikingly, all but two of the TA systems present in *M. tuberculosis* are absent in the closely related pathogen *M. marinum*. Closer analysis of these revealed that the antitoxin gene for one of them, *Rv3181c*, contains two point mutations resulting a significantly truncated, and likely nonfunctional, protein (data not shown). This lack of conservation of TA systems was surprising given that *M. marinum* is thought to be the closest genetic relative of *M. tuberculosis* outside of the MTBC [35],[45]. These findings strongly support the idea that TA gene expansion occurred after the MTBC and *M. marinum* diverged from their last common ancestor, and suggests that these systems play an important role in the unique biology of the MTBC. In support of this idea and consistent with the known origins of TA systems in other bacteria, we discovered that many of the *M. tuberculosis* TA systems are encoded in locations in the genome that were previously identified as regions of horizontal gene transfer [35],[46]. By cross referencing the list of TA systems identified here with previously defined genomic islands [35],[46], we discovered that 24 (37%) of these systems are located in these regions (Table 1). It has been proposed that the acquisition of foreign DNA sequences via horizontal gene transfer was a defining event in the speciation of the MTBC [42],[46],[47]. The large number of TA systems that were acquired during this large influx of heterologous DNA, and subsequently maintained, lends support to the idea that these genes are integral to the biology of the MTBC.

### Inducible expression in *M. smegmatis* identifies many functional TA systems

To begin to understand the functions and biological roles of the numerous TA systems of *M. tuberculosis*, we first sought to determine the number of putative TA systems that encode functional toxins. We used the inducible acetamidase promoter to conditionally express 78 of the putative toxin genes we identified in *M. smegmatis*. We were unable to clone and express 10 putative genes, likely due to differences between our strain (Erdman) and the published sequence of H37Rv. Toxicity was assessed by plating 10-fold dilutions of cultures on solid media in the presence or absence of inducer. Genes encoding a toxic protein product, such as *Rv0301* and *Rv2829c*, inhibited growth of cultures on plates with inducer, but did not affect growth of bacteria on plates without inducer (Figure 1B). This method identified a total of 32 genes that resulted in toxicity when expressed, while the remainder of the genes tested did not inhibit growth under inducing or non-inducing conditions (Table S2). Since many genes can be toxic to cells when over-expressed, it was important to show that expression of the cognate antitoxins of these genes could relieve the toxic activity. Therefore, for each gene that was toxic, we then co-expressed the toxin and antitoxin, under the control of the same inducible promoter, to determine if it allowed cell growth in the presence of inducer (Figure 1B). Toxic proteins that were inactivated by their putative antitoxins, as in the case of Rv0300-0301 and Rv2829c-2830c, were then considered functional TA systems. For two of the RelBE homologs, *Rv1246c* and *Rv2866*, we were unable to obtain transformants in *M. smegmatis*. This result is most likely due to low levels of expression from the acetamidase promoter in the absence of inducer. For these toxins, antitoxin activity was assessed by the ability to obtain transformants with the vector containing both the toxin and the antitoxin.

In total, we discovered 30 pairs of genes in the *M. tuberculosis* genome that function as toxin-antitoxin genes in *M. smegmatis* (Figure 1C and 1D). The majority of the TA systems we identified came from those found by BLAST and PIN-domain containing proteins. Indeed, nearly half of the putative systems identified by these methods are functional TA systems. However, of the genes found by our homology-independent search, a much lower percentage of genes were subsequently found to be functional TA systems. This is not surprising, given that the majority of these genes are not predicted to have protein domains associated with toxin or antitoxin activity. While this method was less efficient, the three novel TA systems identified (*Rv0298-0299*, *Rv0909-0910*, and *Rv2653c-2654c*) are of particular interest. Although Rv0299 appears to be distantly related to MazF, neither Rv0910 nor Rv2653c bear any homology to known TA systems, nor to one another, raising the possibility that they may function by novel mechanisms of toxicity (Figure 1D).

### Comparative genomic analysis reveals a novel, conserved TA system

**Figure 3:**
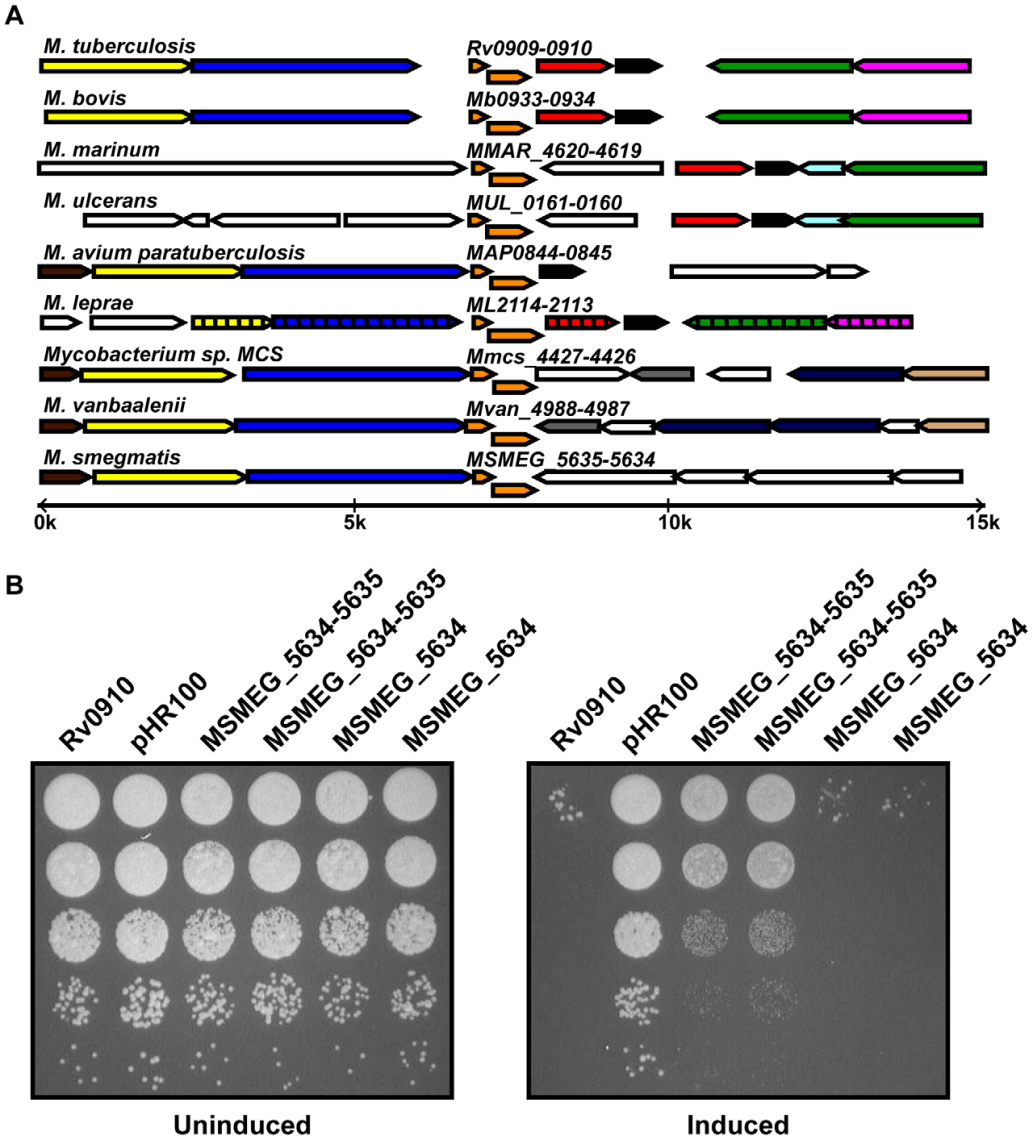
Conservation of a novel TA system. (A) Genomic regions of *Rv0909-Rv0910* TA module orthologs (orange) across diverse mycobacteria showing conservation of toxin and cognate antitoxin genes as well as surrounding genomic context, as indicated by color coding of orthologs across species. (B) *M. smegmatis Rv0909-0910* orthologs encode a functional TA system. *M. smegmatis* cells expressing MSEMG_5634 alone or MSMEG_5634-5635 under the control of the inducible acetamidase promoter were serially diluted and plated on solid media with (right panel) or without (left panel) 0.2% acetamide. (A) adapted from the tree-browser function in MicrobesOnline [60].

Strikingly, *Rv0910* was the only toxin present in all genomes analyzed and, in all cases, orthologs of the putative antitoxin *Rv0909* were also present nearby (Figure 3A). In contrast to many of the other TA systems identified, this operon is not encoded in a genomic island, and its position in the genome is relatively conserved throughout the genus (Figure 3A and Table 1). These findings suggest that the *Rv0909-Rv0910* system may play a conserved role in the physiology of this otherwise diverse group of bacteria.

To determine whether this novel, conserved putative TA system acts as a TA system in other mycobacteria, we cloned the *Rv0909-0910* orthologs *MSMEG_5635-5634* from *M. smegmatis*. We assessed the ability of MSEMG_5634 to inhibit cell growth as well as the ability of MSEMG_5635 to rescue growth inhibition as described above. Plating cells expressing toxin alone on media containing inducer led to growth inhibition that was ameliorated when the cognate antitoxin was co-expressed with the toxin (Figure 3B). These results show that a second member of this novel TA family functions as a TA pair, supporting the idea that *Rv0909-0910* represents the founding member of a new TA family.

### VapB antitoxins show specificity for their cognate VapC toxins

**Figure 4:**
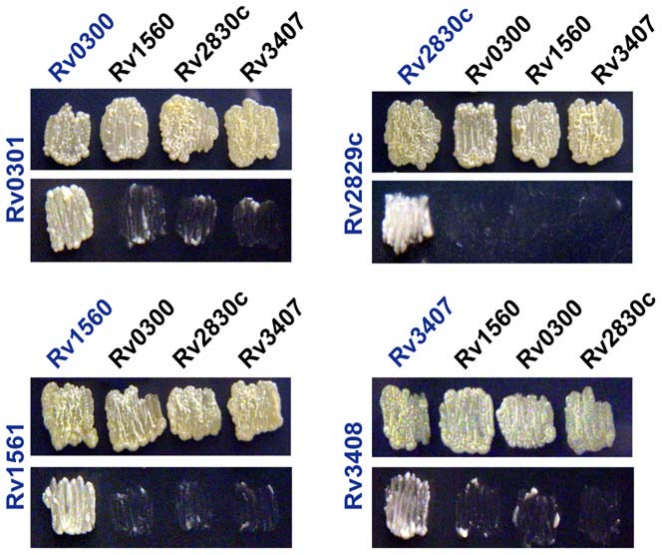
VapB Antitoxins are specific for their cognate VapC toxins. *M. smegmatis* cultures carrying VapC toxins under control of the inducible acetamidase promoter and VapB antitoxins under the control of a tetracycline-inducible promoter were assessed for toxin activity. The VapC toxin being tested is indicated on the left side and the VapB antitoxins are indicated across the top of each set of panels. Each set of panels includes strains tested on solid media without (top) and with (bottom) inducers. The cognate toxin-antitoxin pair for each set is indicated in blue.

The VapBC family comprises, by far, the largest family of TA systems in *M. tuberculosis*. Given that there are a large number of these related genes, we wanted to determine the potential for cross-talk between VapB antitoxins and VapC toxins. Specifically, we wanted to determine if VapB antitoxins can inactivate non-cognate VapC toxins, resulting in the inhibition of toxicity. To address this question we expressed four heterologous VapB and VapC proteins, under the control of separate inducible promoters, and assessed growth in the presence and absence of both inducers. Remarkably, although these are related proteins, our results show that these antitoxins are only able to inhibit their cognate toxins (Figure 4). Although we analyzed only a subset of the VapBC family, this data strongly suggests that VapB antitoxins are highly specific for their associated toxins. Given these results, we conclude that cross-talk is similarly unlikely to occur *in vivo*.

### VapC homologs inhibit translation and have RNase activity *in vitro*

**Figure 5:**
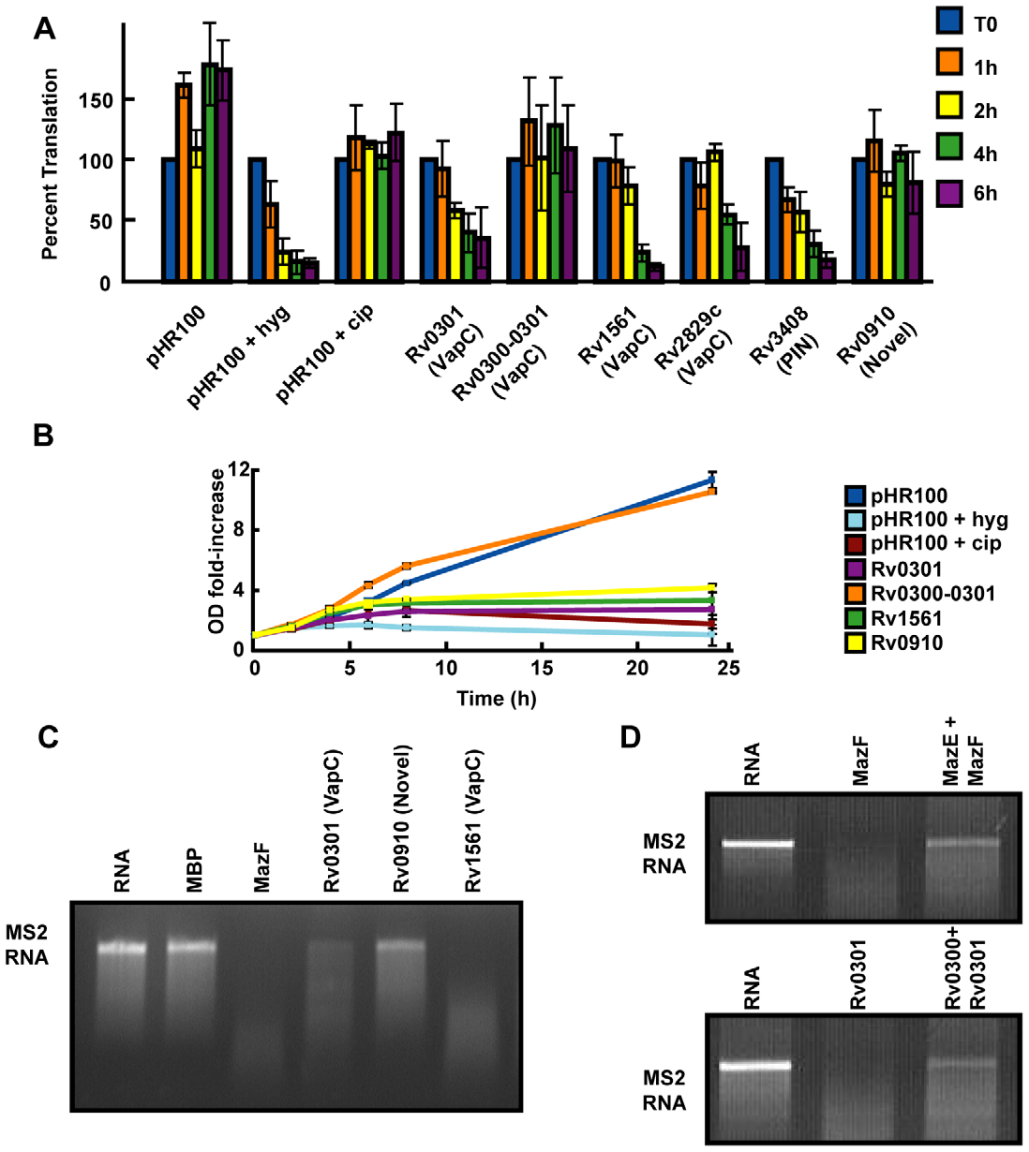
VapC homologs have RNase activity and inhibit translation but a novel toxin does not. (A) Cultures of *M. smegmatis* harboring empty vector (pHR100) or acetamide-inducible toxin constructs were treated with 0.2% acetamide. At the indicated times, cells were labeled with of ^35^S-methionine at 37°C for 1 min and incorporation of radioactivity was measured. Incorporation at t = 0 was set as 100% translation. As controls, cells were treated with 0.5 µg/ml ciprofloxacin (cip), or 25 µg/ml hygromycin (hyg). The average of three experiments is shown and error bars represent the standard deviation. (B) Cultures of *M. smegmatis* were grown and induced as described above. The OD_600_ of each culture was measured at the indicated times. Results are plotted as fold-increase of OD_600_ at each timepoint as compared to OD_600_ at t = 0. The average of three experiments is shown error bars represent the standard deviation. (C) Purified toxins were incubated with MS2 RNA for 3 h at 37°C. The RNA was then purified and electrophoresed in a 1% denaturing agarose gel. Included as controls were RNA alone (RNA), His-MBP (MBP), and *E. coli* MazF (MazF). (D) Purified toxins MazF and Rv0301 were incubated with their respective GST-tagged antitoxins MazE (10 µg) and Rv0300 (5 µg) and 0.8 µg MS2 RNA for 3 h at 37°C. Reactions were electrophoresed in a 2% agarose gel.

Many toxins of TA systems function as RNases and result in translation inhibition when activated [14],[48]. In particular, PIN domain-containing proteins, including some VapC homologs, have been shown to have RNase function [17]–[19],[49]. We expressed the VapC homolog Rv0301 in *M. smegmatis* and monitored bulk translation via incorporation of ^35^S-methionine over a six hour time course. As shown in Figure 5A, Rv0301 expression led to inhibition of translation, an effect that was reversed by co-expression of its antitoxin, Rv0300. The effect on protein synthesis preceded the inhibition of growth caused by this toxin (Figure 5B). Likewise, expression of three other VapC homologs (Rv1561, and Rv2829c, Rv3408) also inhibited translation (Figure 5A). As a control, addition of hygromycin, an antibiotic that targets protein synthesis, inhibited incorporation of ^35^S-methionine (Figure 5A) and growth (Figure 5B), as early as one hour after addition to the media. The modest difference in the kinetics of translation inhibition between toxin induction and addition of antibiotics is likely due to time required for transcription and synthesis of the toxin.

Given the proposed ribonuclease function associated with PIN domains, we reasoned that a likely mechanism for translation inhibition of the toxins tested was RNA cleavage. Indeed, *in vitro* RNase activity of an *M. tuberculosis* VapC homolog was recently demonstrated [49]. To test the ability of these proteins to hydrolyze RNA, we performed *in vitro* RNA cleavage assays with purified VapC proteins and the viral MS2 RNA, a substrate that has been effectively used to detect RNase activity [13]. As shown in Figure 5C, incubation of MS2 RNA with *E. coli* MazF, or with either of two *M. tuberculosis* VapC homologs, Rv0301 and Rv1561, resulted in degradation of the RNA, though Rv0301 exhibited less potent RNase activity. The incubation of the toxins MazF and Rv0301 with their cognate antitoxins, MazE and Rv0300, respectively, inhibited this RNase activity (Figure 5D). As controls, we also included MS2 RNA incubated with buffer alone, as well as an MBP-His protein fragment, which both failed to cleave MS2 RNA (Figure 5C). These results show that *M. tuberculosis* VapC homologs inhibit translation and strongly suggest that these toxins affect translation directly via RNA cleavage.

### A novel toxin inhibits growth but not translation

In contrast to our results with the VapC homologs, expression of the novel toxin Rv0910 did not result in inhibition of translation throughout the duration of the assay (Figure 5A). However, expression of this toxin did inhibit cell growth, suggesting that Rv0910 targets a cellular process other than translation (Figure 5B). Additionally, incubation of MS2 RNA with purified Rv0910 yielded intact MS2 RNA, consistent with this idea. The specificity of the translation assay was verified by treating cultures with the antibiotic ciprofloxacin, a DNA replication inhibitor. Although the antibiotic inhibited growth (Figure 5B) and caused DNA damage as measured by *recA* expression (Figure S2), it did not affect translation over the course of the experiment (Figure 5A). This important specificity control shows that disruption of other macromolecular synthesis pathways does not affect translation during this assay, and is similar to the results obtained with expression of Rv0910. Taken together, these results strongly suggest that Rv0909-0910 represents a novel TA system that inhibits cell growth via a mechanism distinct from the VapBC family.

### Subsets of TA systems are expressed in *M. tuberculosis* under conditions of stress

The presence of such a large number of TA systems presents an obvious question: What is the benefit of having so many of these genes in one organism? We postulated that subsets of TA systems important for *M. tuberculosis* biology may respond to different cellular stresses. To test this hypothesis, we determined if any of the functional TA systems in *M. tuberculosis* were transcriptionally activated under two conditions encountered during infection. In particular, we examined the response of TA systems under hypoxic conditions in culture and during infection of IFN-γ-stimulated murine bone marrow-derived macrophages. To assess TA activation, we took advantage of the fact that most antitoxins also function as transcriptional autorepressors, and thus degradation of an antitoxin results in increased transcription of its operon. Although this increase in transcription leads to increased protein synthesis of both the toxin and antitoxin, the unstable antitoxin is typically selectively targeted for degradation by a protease, further increasing transcription of the operon, while the more stable toxin interacts with its cellular target [48]. In this manner, we are using the increase in transcription as an indirect read-out of TA system activation via degradation of the antitoxin, thus relieving its transcriptional inhibitory activity.

**Figure 6:**
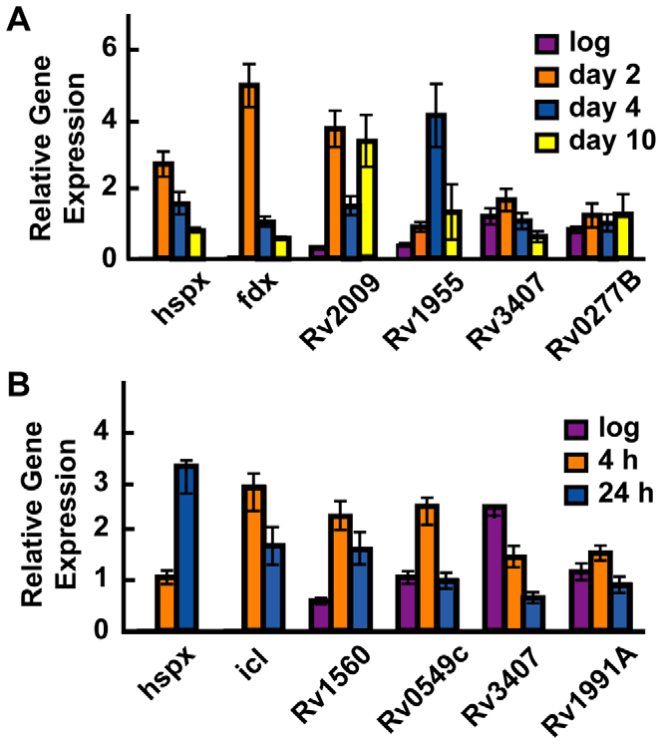
Subsets of TA systems are activated during stress. (A) Cultures were grown in 1 liter roller bottles with a headspace ratio of 0.5 (830 ml culture) at 37°C, with slow stirring to induce NRP. At the indicated days, cells were collected and RNA was isolated and amplified. Gene expression was measured using qPCR. One of three similar experiments is shown and error bars represent the standard deviation within this experiment. (B) IFN-γ stimulated bone marrow-derived macrophages were infected with *M. tuberculosis* at an MOI of 10. Bacterial RNA from intracellular bacilli was isolated and amplified. Gene expression was measured by qPCR and normalized to 16S rRNA as an internal control. Gene expression from *in vitro* grown log-phase cultures (log) is included for comparison. One of three similar experiments is shown and error bars represent the standard deviation within this experiment.

We monitored the expression of each of the 30 functional TA systems by quantitative PCR and obtained detectable signal for 25 TA systems during hypoxia and for 23 systems during macrophage infection (Table S4 and Table S5). Two TA systems, *Rv2009-2010* and *Rv1955-1956*, were induced during hypoxia (Figure 6A). Transcription of *hspX* and *fdxA*, two genes belonging to the dormancy regulon that are very highly induced during hypoxia, was also induced, demonstrating that the bacteria experienced a hypoxic environment (Figure 6A). Of the TA systems monitored during macrophage infection, *Rv1560-1561* and *Rv0549c-0550c* were induced. As controls, transcription of *hspX* and *icl* were greatly induced, consistent with previous results of the transcriptional response following macrophage infection [50] (Figure 6B). It is interesting that the two TA systems that are activated during hypoxia are not activated during macrophage infection, given the effectively hypoxic environment generated via nitric oxide signaling following IFN-γ stimulation of the macrophages. Indeed, these two conditions both result in induction of the dormancy regulon [50],[51]. However, the TA genes tested here are not part of the DosR dormancy regulon and are likely activated by independent mechanisms. Certainly, it is possible that other TA systems are also activated under these conditions but this was not detectable by the methods used here. In light of these data, it appears that TA systems are regulated independently from one another, and expression of at least four of these TA systems is modulated in response to different environmental stresses. This supports our hypothesis that specific subsets of TA genes are regulated in response to changes in the environment.

## Discussion

Here we have shown that of 88 putative *M. tuberculosis* toxin-antitoxin loci, 30 encode functional TA systems. The numbers of both the putative and functional TA systems are significantly greater than in any other organism studied thus far. Our sequence-based searching of the *M. tuberculosis* genome, in conjunction with previous bioinformatic approaches, has identified 62 TA systems with homology to known TA systems [16],[32],[33]. Because mycobacterial TA genes may have limited sequence identity with distantly related homologs from other bacteria, there may be additional TA systems that have yet to be discovered. Importantly, incorporation of our sequence-independent method identified an additional 26 loci bearing no homology to known TA genes, allowing us to assemble one of the most comprehensive lists of putative TA systems in *M. tuberculosis* to date.

It is striking that nearly all of the TA systems we identified are well conserved among the MTBC but are largely absent from species outside of this complex, including *M. marinum*. The paucity of TA systems in *M. marinum* is particularly notable as the two bacteria are highly related, sharing 3000 orthologs with an average amino acid identity of 85% [35]. Therefore, the massive expansion of TA systems is a distinguishing feature of the MTBC, and the acquisition and maintenance of these genes was likely instrumental for the evolution of *M. tuberculosis*. We discovered that of the 423 protein-coding genes located within these regions, 48 are TA genes (Table 1). This frequency of TA genes (11%) is significantly higher than that of the entire *M. tuberculosis* genome (4%), indicating that there is an enrichment of TA loci in these regions. Our results, combined with data from Becq *et al.* [46] and Stinear *et al.* [35], lends strong support to the idea that many of the TA systems were recently acquired via horizontal gene transfer after the divergence between *M. tuberculosis* and *M. marinum*. It is possible, however, that some of these genes were acquired in a common ancestor of the slow-growing mycobacteria and subsequently lost in a subset of extant species. In support of the latter hypothesis, *M. leprae* and *M. kansasii*, bacteria that are thought to be more distantly related to the MTBC, contain TA system orthologs that are clearly absent from *M. marinum* (Figure 2 and Table S3). Alternatively, the assignment of *M. marinum* as the closest relative of the MTBC may be incorrect, as supported by whole-genome comparisons that place *M. kansasii* as the nearest neighbor of the MTBC [43]. Given the evidence that the vast majority of TA systems have no counterparts in mycobacteria outside of the MTBC, the most parsimonious explanation for their expansion is that these genes were acquired after speciation. In addition, further amplification of TA systems may have occurred in the MTBC via gene duplication events.

It is curious that so many TA loci are present in the *M. tuberculosis* genome. One possible reason for this expansion is that genomic TA systems may function to stabilize the *M. tuberculosis* chromosome, akin to the role of TA systems in stabilizing plasmids [52]. Since toxins are typically more stable than antitoxins, TA systems inhibit post-segregational plasmid loss because cells that do not inherit the episome rapidly deplete the antitoxin protein, leading to toxin activation and inhibition of growth. Indeed, there is recent evidence that chromosomal TA systems may protect adjacent regions of the chromosome from deletion [9]. In support of this notion, many of the TA systems identified here are encoded on genomic islands that include genes important for *M. tuberculosis* virulence or physiology (Table 1). Alternatively, TA systems may participate more directly in the physiology of *M. tuberculosis* by functioning as stress response elements, as has been demonstrated in *E. coli* [2],[3],[48],[53]. There is a growing body of evidence that some TA systems are induced during exposure to adverse environmental conditions, such as exposure to antibiotics, allowing cells to respond and adapt to the assault [54]. Indeed, a screen to identify mutants in *M. tuberculosis* with altered growth kinetics during transitions in carbon availability revealed numerous TA systems identified in our analysis likely participate in growth rate decisions [55]. In addition to responding to stress directly, the HipBA TA system participates in generating slowly or non-replicating persister cell subpopulations within a larger group of growing bacteria [7]. In this way, TA systems provide resistance to non-favorable environmental conditions as persister cells are better able to survive the onslaughts of severe stress than are replicating bacteria. Given the number and diversity of TA systems in *M. tuberculosis*, it is possible that some serve to stabilize the genome while others serve to provide stress resistance.

Of particular interest are TA systems that participate in the biology of *M. tuberculosis*, either participating in stress-response or persister formation. Our findings that four TA systems are activated during cellular stress supports the notion that these loci participate directly in *M. tuberculosis* physiology. For example, both *Rv1955-1956* and *Rv2009-2010* are induced during the transition to hypoxia (Figure 6A), suggesting they play a role in the adaptation of *M. tuberculosis* to low oxygen conditions. Curiously, both of the hypoxia-induced TA loci, *Rv1955-1956* and *Rv2009-2010*, are located within the same genomic island [35]. Although these genes are not part of the “dormancy regulon”, notable members of this regulon, *dosT* and *fdxA*, are also in the same genomic island. It may be that acquisition of this entire region helped promote *M. tuberculosis*' ability to respond to a hypoxic environment. In addition to the TA genes upregulated by hypoxia, *Rv1560-Rv1561* and *Rv0549c-0550c* are specifically upregulated during macrophage infection (Figure 6B), in agreement with previous studies suggesting that these genes may be important during infection [56],[57]. Taken together, these data suggest that a subset of TA systems is important for *M. tuberculosis in vivo*, perhaps as stress-response elements.

In an attempt to identify novel classes of TA systems, we incorporated a sequence-independent method in our bioinformatics search. Importantly, this strategy revealed three novel TA systems. One of these, Rv0909-0910, is conserved among the diverse range of the mycobacterial species analyzed (Figure 2 and Figure 3A), suggesting an ancient history and likely fundamental role in mycobacterial physiology. Our results indicate that Rv0910 lacks RNase activity (Figure 5C) and thus probably functions by an alternate mechanism than the majority of *M. tuberculosis* TA systems. Interestingly, Rv0910 is most similar to the polyketide cyclase group of the START domain superfamily of proteins [58]. Although it is not clear how a polyketide cyclase would function to inhibit cell growth, our results demonstrate for the first time that these two genes have toxin and antitoxin activities. It is interesting to note that mycobacteria encode numerous polyketide synthases and have an enormous capacity for lipid synthesis [35]. Since many TA toxins inhibit macromolecular synthesis (translation, DNA synthesis), it is tempting to speculate that Rv0910 may inhibit lipid biosynthesis.

Although our approach yielded a total of 30 functional TA systems, it is likely that there are additional TA systems in the *M. tuberculosis* genome. Our search for putative systems was biased by using characteristics of known TA systems, including constraints on size and gene organization. Therefore, any TA system with different features would have been excluded from our search. Indeed, two other groups have incorporated homology-independent methods for comprehensive discovery of TA systems in microbial genomes and identify an additional 12–44 putative systems in the *M. tuberculosis* genome, depending on the level of scoring confidence chosen (Figure S1). We also cannot rule out the possibility that some of the toxins that did not inhibit growth were simply not expressed at sufficiently high levels using our system. Additionally, although *M. smegmatis* and *M. tuberculosis* are similar, we cannot discount the possibility that there are some species-specific factors that may be required for the proper function of some of the TA systems tested.

Our results differ significantly from two recent reports in which *M. tuberculosis* TA systems were expressed in *E. coli* [12],[39]. Of the 78 putative systems we tested in *M. smegmatis*, only 38 have been evaluated in *E. coli*. Of the 30 functional TA systems we identified, seven were not functional in *E. coli* and a further 40 putative systems were not assessed. In contrast, only four systems were uniquely toxic in *E. coli*. The different results obtained in these studies may be due to the use of a distantly related host, *E. coli*, rather than the more closely related species, *M. smegmatis*. For example, specific factors such as transcript GC content, may greatly influence the number of potential targets of an RNase, and these differences are minimized by using another mycobacterial species.

**Figure 7:**
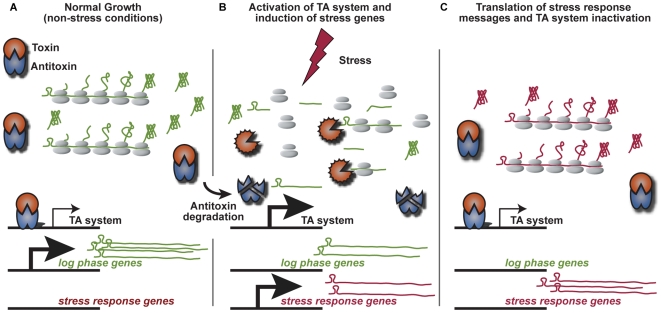
Model of stress-induced TA system activation and proteome remodeling. (A) Under normal growth conditions, the antitoxin is bound to its cognate toxin, this complex in turn binds its own promoter, inhibiting transcription. (B) In response to stress, the antitoxin is specifically degraded, releasing the toxin to cleaves existing transcripts. Additionally, degradation of the antitoxin results in increased transcription of the TA system. (C) Upon stabilization of the antitoxin, the TA system is inactivated and toxin-antitoxin complex, resulting in transcriptional inhibition of its operon. This pulse of toxin activity functionally erases the previous transcriptional profile allowing the newly stress induced messages to be preferentially translated.

Given the huge expansion of VapBC homologs in *M. tuberculosis*, it seems likely that there are many more toxins that function via RNA cleavage than by other mechanisms. It is perplexing that one genome would have so many genes encoding proteins that perform the same function. One possibility is that most of these TA systems participate solely in genome stability and thus redundant function would not be an issue. However, all four of the stress-responsive TA systems we identified are putative RNAses. Therefore, another possibility is that, although they have the same function, specific TA systems are under different regulatory controls, such that only a subset are activated in response to particular stresses. Having a wide variety of mRNases under diverse regulatory mechanisms would allow the cell to adapt to many different conditions. An alternative explanation is that the different mRNases are actually functionally distinct. Under certain conditions, it may be useful for the cell to express an mRNase with a short, ubiquitous cleavage site that will target most of the existing messages in the cell, and allow for newly transcribed messages to be translated. This provides an efficient way to erase the previous transcriptional profile of the bacterium, allowing the cell to reprogram the proteome and thus rapidly change the metabolic state of the cell during conditions of stress (Figure 7). Alternatively, some TA systems may target only a limited number of messages and, therefore, not inhibit bulk translation. For example, one possible mechanism to tailor the response of the cell upon expression of an mRNase is through the recognition site at which mRNA cleavage occurs. Indeed, the cleavage sites for two *M. tuberculosis* MazF homologs target pentad sequences, longer than the three-residue recognition site of MazF in *E. coli* [13]. This may allow the targeting of specific messages, giving rise to more subtle changes in the metabolic state of the cell. It may be the presence of so many mRNases that allows *M. tuberculosis* to regulate growth, survival and metabolism during a wide range of environmental stresses, including those encountered during infection. This hypothesis is consistent with our findings that the vast majority of TA systems are present only in the virulent mycobacteria of the MTBC.

## Materials and Methods

### Strains and plasmids

All strains, plasmids and primers used in this study can be found in Table S6.

### Identifying putative TA systems

TA system homologs in *M. tuberculosis* were identified using PSI-BLAST (NCBI) with a cut-off E-value of 10^−2^. Iterations were repeated until we obtained no new hits below the cut-off E-value. The input sequences for this analysis included the toxins and antitoxins of 8 major TA system families [32]. The origins of the input sequences of each TA system family used for BLAST analysis were as follows: CcdBA (F plasmid from *E. coli*), MazEF (MazEF from *E.coli*, PemK from *Rhodococcus erythropolis*), Doc/Phd (enterobacteria phage P1, Phd from *Frankia alni* ACN14a), RelBE (RelBE from *E. coli*, PasBA from plasmid pTCF14 of *Acidithiobacillus caldus*, YoeB/YefM from *E. coli*), HipBA (HipBA from *E. coli*), ParDE (ParDE from plasmid RK2 of *E. coli*), HigBA (HigBA from plasmid Rts1 of *E. coli*, HigA of *Xylanimonas cellulosilytica* DSM 15894), VapBC (VapBC from *Frankia sp.*, StbBC from plasmid pDC3000B of *Pseudomonas syringae*). Following identification of toxin genes we determined if an adjacent upstream gene smaller than the putative toxin gene was present. Following identification of antitoxin genes we determined if an adjacent downstream gene larger than the putative toxin was present. In both cases, we required a maximum distance of 150 bp between the putative toxin and antitoxin. Homologs for which we were unable to find an adjacent cognate toxin or antitoxin or in which the adjacent gene did not meet our criteria for either size or distance between genes were excluded. In cases where the putative toxin and antitoxin of an adjacent pair were homologous to different TA system families, we assigned the pair based on the homology of the toxin gene. PIN domain-containing proteins were identified as previously described [16]. To find novel TA pairs we searched the *M. tuberculosis* genome for pairs of genes as previously described [40]. In summary, we identified pairs of open reading frames (ORFs) encoding hypothetical proteins in the *M. tuberculosis* genome of less than 150 amino acids, were less than 150 bp apart, and in which the upstream ORF was smaller than the downstream ORF.

### Comparative genomics study

The fully sequenced and annotated genomes were downloaded from the NCBI database. The latest assemblies of unfinished genomes were downloaded from the Sanger Institute website with permission from Dr. Julian Parkhill. or the NCBI database with permission from Dr. Marcel Behr. We used standard BLASTP or TBLASTX to search protein or nucleic acid databases of each genome for homologs of the *M. tuberculosis* toxin proteins identified in this study. For each toxin homolog or ortholog identified, we determined whether an adjacent putative antitoxin was present. Orthologs were defined as BLAST reciprocal best hits (E-value<10^−6^) displaying conserved synteny with other orthologs [46]. A detailed description of the phylogenetic analysis can be found in Text S1 and Table S3.

### Assessing toxin and antitoxin activity

All putative *M. tuberculosis* toxin genes and toxin-antitoxin gene pairs were inserted downstream of the inducible acetamidase promoter in plasmid pHR100. Toxin and antitoxin activity was assessed by growing *M. smegmatis* carrying the appropriate vector at 37°C on 7H10 solid media with 0.2% Tween-80, 25 µg/ml kanamycin, and 0.2% acetamide to induce gene expression. Growth was assessed after three days of incubation. Cross-talk between non-cognate VapB and VapC proteins was assessed by co-transforming *M. smegmatis* with the VapC toxin under the control of the acetamidase promoter and the VapB antitoxins under the control of the tetracycline-inducible promoter in plasmid pUV15tetORm [59]. Toxicity was assessed by patching colonies onto solid media in the absence (7H10 with 0.2% Tween-80, 25 µg/ml kanamycin, 50 µg/ml hygromycin) or presence of both inducers (7H10 with 0.2% Tween-80, 25 µg/ml kanamycin, 50 µg/ml hygromycin, 0.2% acetamide, 50 ng/ml ATc).

### Translation assays

*M. smegmatis* cells carrying the appropriate expression vector were grown to early log phase at 37°C in 7H9 Middlebrook media with 25 µg/ml kanamycin. At OD_600_ 0.3, acetamide was added to a final concentration of 0.2% to induce gene expression. Control cultures were treated with either 0.5 µg/ml ciprofloxacin or 25 µg/ml hygromycin. Samples of 2 ml were harvested by centrifugation at the timepoints indicated and resuspended in 0.5 ml media containing 5 µCi of ^35^S-methionine. After one minute of incorporation at 37°C, reactions were stopped by adding 1 ml 40 mM sodium azide and immediately frozen in liquid nitrogen. Proteins were precipitated with 10% trichloroacetic acid and concentrations were determined using Micro BCA Protein Assay Kit (Pierce). Radioactivity incorporated was assessed via a liquid scintillation counter and normalized to protein concentration in each sample. The radioactivity incorporated at t = 0 for each culture was set as 100% translation and all subsequent measurements were compared to this value.

### Purification of proteins

Toxin and antitoxin proteins for the RNase assay were expressed in *E. coli* BL21 (DE3) *pLysS* cells. The toxins were expressed as N-terminal (His)_6_-MBP-TEV tagged fusions while the antitoxins were expressed as N-terminal GST fusions. Protein expression was induced for 3 h at 37°C with 500 µM IPTG. Toxin proteins were purified using Talon metal affinity resin (Clontech). The resin was washed using buffer (50 mM NaPO_4_, 800 mM NaCl, pH 7.1) containing imidazole at concentrations of 20, 40, and 60 mM and eluted using 250 mM imidazole. Antitoxin protein were purified using glutathione resin, washed with 30 column volumes of the buffer indicated above and eluted using 15 mM reduced glutathione. Proteins were subsequently dialyzed in buffer containing 50 mM NaCl and 25 mM Tris-HCl. Toxin proteins were TEV-digested at a ratio of 1∶25 (TEV∶protein) in buffer containing 50 mM NaCl, 2 mM Tris-HCl and 2 mM DTT to remove the tags.

### RNase activity

1.6 µg MS2 RNA (Roche) was incubated for 3 h with 1 µg of each purified protein at 37°C in 10 mM Tris-HCl (pH 7). RNA was purified and samples were heated to 95°C for 5 min and placed on ice for 1 min before loading in a denaturing agarose gel (1% agarose, 6.5% formaldehyde, 1× MOPS buffer). To assess antitoxin activity, 1 µg of purified toxin protein was incubated with antitoxins Rv0300-GST (5 µg) or MazF-GST (10 µg). RNA from each reaction was electrophoresed in a 2% agarose gel under non-denaturing conditions.

### *In vitro* hypoxia

NRP was induced essentially as described in [27] with a larger culture volume (830 ml) in 1 liter roller bottles to achieve a headspace ratio of 0.5. Bacteria were pelleted and lysed at the indicated timepoints by bead beating with 200 µl 0.1 mm zirconia beads (Biospec) at maximum speed for 90 s in 1 ml Trizol and total RNA was isolated via chloroform extraction and sodium acetate precipitation as previously described. [29] Bacterial RNA was amplified using the MessageAmp II Bacteria Prokaryotic RNA Kit per the manufacturer's instructions (Ambion).

### Macrophage infections

Bone marrow-derived macrophages were isolated from C57BL/6 mice and cultured for 6 d in media containing 30% L-cell supernatant in the presence of antibiotics. Macrophages were stimulated with recombinant mouse IFN-γ at a final concentration of 50 units/ml for 24 h prior to infection. Macrophages were infected using DMEM containing 10% horse serum at a multiplicity of infection of 10, incubated for 2 h, washed and fresh medium was added. At the indicated timepoints, *M. tuberculosis* RNA from inside macrophages was isolated and amplified as previously described [50].

### Quantitative PCR

*M. tuberculosis* and *M. smegmatis* from *in vitro*-grown log-phase cultures were pelleted and lysed by bead beating in Trizol as described above and total RNA was isolated [29] and used for quantitative real-time PCR (qPCR) with the oligonucleotides specified (Table S7). The cDNA used for qPCR was generated with 3 µg of total RNA using the Superscript III First Strand Synthesis for RT-PCR kit (Invitrogen). Standard curves were generated by measuring the concentration of each message in a reference sample of RNA pooled from all indicated conditions and timepoints analyzed for each experiment. All values reported are given as relative expression of each gene compared to 16S RNA (gene/16S).

## Supporting Information

Figure S1 Venn diagrams illustrating the relationships between putative TA systems identified by three different algorithms utilizing combined homology-dependent and independent methods for finding TA loci in microbial genomes. Only predictions of complete TA pairs are included in this analysis and genes with multiple predicted partners are included only once. (A) Comparison between putative TA systems identified here, in Makarova, *et al.* [9] and by RASTA-Bacteria [10] using a strict RASTA-Bacteria cutoff score of >70%. (B) Comparison between putative TA systems identified here, in Makarova, *et al.* [9] and by RASTA-Bacteria [10] using a strict RASTA-Bacteria cutoff score of >55%. Gene lists and Venn diagram figures were generated using the web-based tools Venn Diagram Generator (http://www.pangloss.com/seidel/Protocols/venn.cgi) and Wybiral's Venn Diagram Generator (http://davy.wybiral.googlepages.com/venn.html), respectively.
(0.28 MB TIF)

Figure S2 *recA* is induced after treatment with ciprofloxacin. *M. smegmatis* harboring pHR100 was grown to early log phase and treated with 0.5 µg/ml ciprofloxacin. At 0, 2, 4, and 6 h, 2 ml aliquots were taken and RNA was harvested. The expression of *recA* was measured by qPCR. Three replicates are shown.
(0.18 MB TIF)

Table S1 BLAST analysis to identify *M. tuberculosis* TA system homologs. Toxin and antitoxin system homologs used for PSI-BLAST analysis, *M. tuberculosis* BLAST hits and E-values are shown. For each homolog identified, we determined if an adjacent putative toxin/antitoxin was nearby and conserved domains present in these proteins are indicated. *M. tuberculosis* homologs that were significantly larger than 150 amino acids were excluded.
(0.03 MB XLS)

Table S2 Results of putative TA system testing. Putative toxin genes, along with the method of identification and toxicity results in *M. smegmatis* are shown. For genes that inhibited growth, the putative antitoxin was co-expressed. These genes were scored as those that relieved toxicity, and are part of a functional TA system (yes), and those that did not (no). The putative antitoxins that were used for testing are indicated in parentheses.
(0.13 MB DOC)

Table S3 Conservation of TA systems across the genus *Mycobacterium*. The top BLAST hit in each organism is shown along with conserved antitoxin (if present). Reciprocal indicates whether the BLAST hit was the top reciprocal hit to the *M. tuberculosis* query toxin and synteny indicates whether the BLAST hit resides in a similar genomic context as the *M. tuberculosis* query toxin. For all strains other than *M. leprae*, top BLASTP hit is shown and top TBLASTX hit is shown for *M. leprae*. Putative antitoxin sequences shaded in grey indicate truncated sequences that are likely nonfunctional. Note that there is a possible duplication of *Rv3384c* in the *M. canetti* genome, but because the assembly used in this analysis is preliminary, this apparent duplication event may be due to an error in the draft assembly.
(0.11 MB XLS)

Table S4 Expression results for all genes tested during hypoxia. Results of qPCR for each *M. tuberculosis* gene tested in two experiments after induction of NRP (hypoxia). Data is expressed as gene/16S and the standard deviation (SD) at each timepoint is shown. Timepoints at which we were unable to detect signal for a given gene are indicated (ND).
(0.12 MB DOC)

Table S5 Expression results for all genes tested during macrophage infection. Results of qPCR for each *M. tuberculosis* gene tested in two experiments at 4 and 24 h after infection of IFN-γ-stimulated wild-type macrophages. Data is expressed as gene/16S and the standard deviation (SD) at each timepoint is shown.
(0.13 MB DOC)

Table S6 Vectors, plasmids, and primers used in this study.
(0.20 MB DOC)

Table S7 Primers used for qPCR of toxin, antitoxin, and control genes.
(0.05 MB DOC)

Text S1 Supporting methods.
(0.04 MB DOC)
