# Supplementary figures and images for: Comprehensive Functional Analysis of Mycobacterium tuberculosis Toxin-Antitoxin Systems: Implications for Pathogenesis, Stress Responses, and Evolution
Source: PLoS Genet. 2009 Dec 11;5(12):e1000767. doi: 10.1371/journal.pgen.1000767 (PMC2781298; doi:10.1371/journal.pgen.1000767)

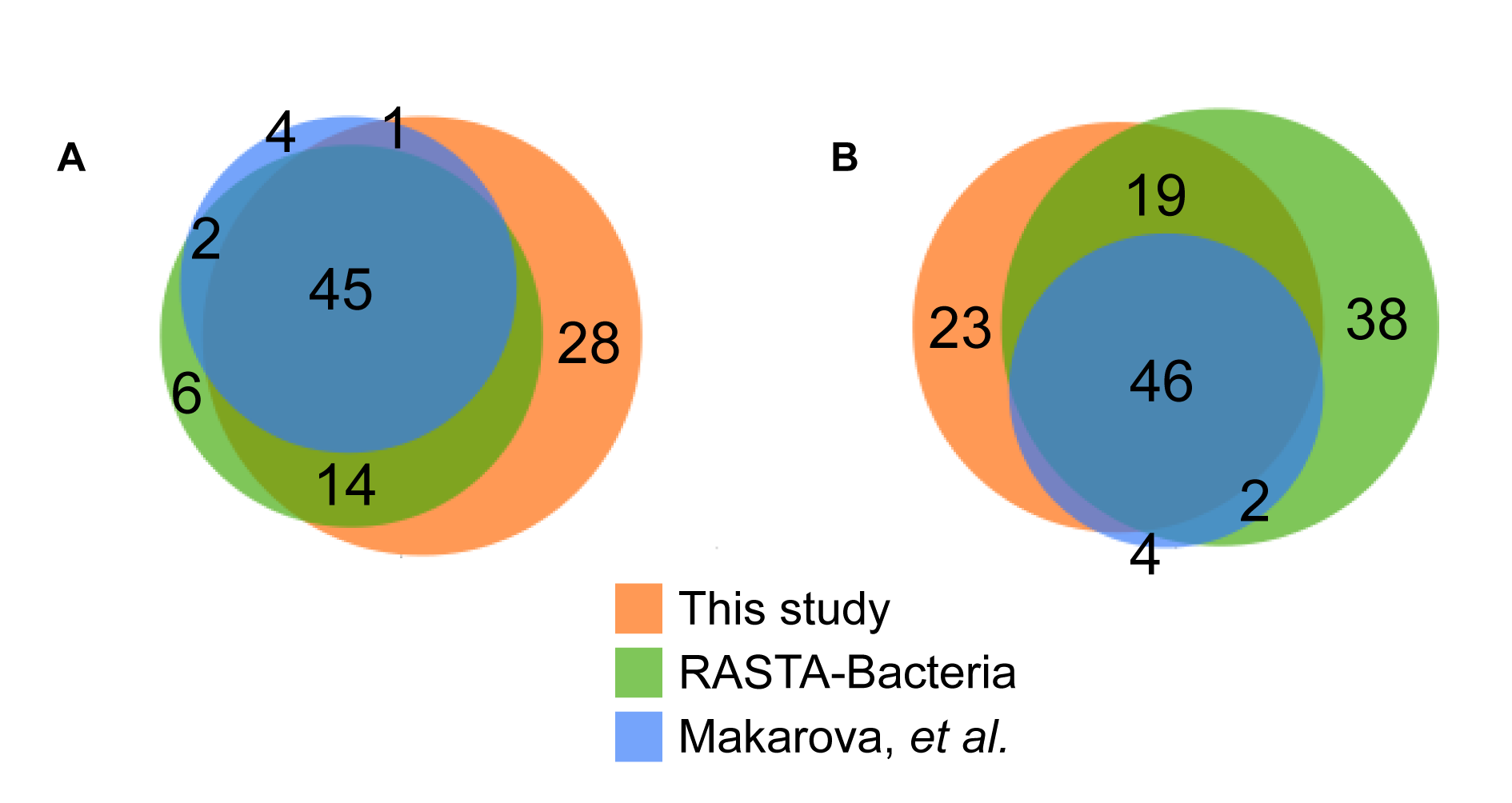

Supplement: Figure S1 — Venn diagrams illustrating the relationships between putative TA systems identified by three different algorithms utilizing combined homology-dependent and independent methods for finding TA loci in microbial genomes. Only predictions of complete TA pairs are included in this analysis and genes with multiple predicted partners are included only once. (A) Comparison between putative TA systems identified here, in Makarova, et al. [9] and by RASTA-Bacteria [10] using a strict RASTA-Bacteria cutoff score of >70%. (B) Comparison between putative TA systems identified here, in Makarova, et al. [9] and by RASTA-Bacteria [10] using a strict RASTA-Bacteria cutoff score of >55%. Gene lists and Venn diagram figures were generated using the web-based tools Venn Diagram Generator (http://www.pangloss.com/seidel/Protocols/venn.cgi) and Wybiral's Venn Diagram Generator (http://davy.wybiral.googlepages.com/venn.html), respectively. (0.28 MB TIF) [file pgen.1000767.s001.tif]

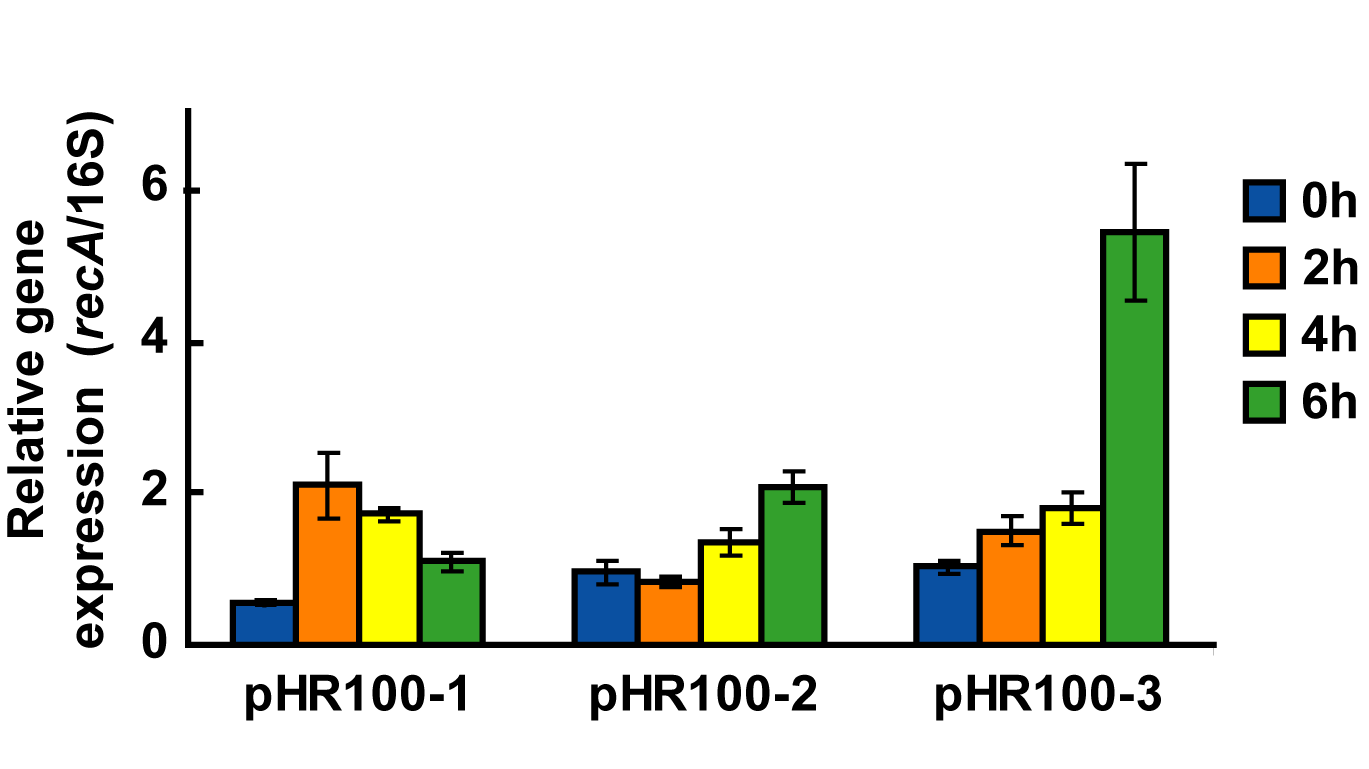

Supplement: Figure S2 — recA is induced after treatment with ciprofloxacin. M. smegmatis harboring pHR100 was grown to early log phase and treated with 0.5 µg/ml ciprofloxacin. At 0, 2, 4, and 6 h, 2 ml aliquots were taken and RNA was harvested. The expression of recA was measured by qPCR. Three replicates are shown. (0.18 MB TIF) [file pgen.1000767.s002.tif]
