# Supplementary material for: Comprehensive Functional Analysis of Mycobacterium tuberculosis Toxin-Antitoxin Systems: Implications for Pathogenesis, Stress Responses, and Evolution
Source: PLoS Genet. 2009 Dec 11;5(12):e1000767. doi: 10.1371/journal.pgen.1000767 (PMC2781298; doi:10.1371/journal.pgen.1000767)
Supplement: Table S2 — Results of putative TA system testing. Putative toxin genes, along with the method of identification and toxicity results in M. smegmatis are shown. For genes that inhibited growth, the putative antitoxin was co-expressed. These genes were scored as those that relieved toxicity, and are part of a functional TA system (yes), and those that did not (no). The putative antitoxins that were used for testing are indicated in parentheses. (0.13 MB DOC) [file pgen.1000767.s004.doc]

| **Protein** | **Method of identification** | **Toxic activity in *M. smegmatis*** | **TA system (antitoxin)** |
| --- | --- | --- | --- |
| Rv0065 | VapC homolog, PIN domain | No |  |
| Rv0240 | VapC homolog, PIN domain | No |  |
| Rv0277c | VapC homolog, PIN domain | Yes | Yes (Rv0277A) |
| Rv0299 | Novel | Yes | Yes (Rv0298) |
| Rv0301 | VapC homolog, PIN domain | Yes | Yes (Rv0300) |
| Rv0456A | MazF homolog | No |  |
| Rv0477 | Novel | No |  |
| Rv0543c | Novel | No |  |
| Rv0549c | VapC homolog, PIN domain | Yes | Yes (Rv0550c) |
| Rv0582 | VapC homolog, PIN domain | Yes | Yes (Rv0581) |
| Rv0595c | VapC homolog, PIN domain | No |  |
| Rv0598c | VapC homolog, PIN domain | No |  |
| Rv0609 | VapC homolog, PIN domain | Yes | Yes (Rv0608) |
| Rv0617 | VapC homolog, PIN domain | No |  |
| Rv0624 | VapC homolog, PIN domain | Yes | Yes (Rv0623) |
| Rv0627 | VapC homolog, PIN domain | No |  |
| Rv0656c | VapC homolog, PIN domain | No |  |
| Rv0659c | MazF homolog | No |  |
| Rv0661c | VapC homolog, PIN domain | No |  |
| Rv0665 | VapC homolog, PIN domain | No |  |
| Rv0749 | VapC homolog, PIN domain | Yes | Yes (Rv0748) |
| Rv0760c | Novel | No |  |
| Rv0910 | Novel | Yes | Yes (Rv0909) |
| Rv0948c | Novel | No |  |
| Rv0960 | VapC homolog, PIN domain | No |  |
| Rv1102c | MazF homolog | Yes | Yes (Rv1103c) |
| Rv1114 | VapC homolog, PIN domain | Yes | Yes (Rv1113) |
| Rv1242 | VapC homolog, PIN domain | Yes | Yes (Rv1241) |
| Rv1246c | RelE homolog | Yes | Yes (Rv1247c) |
| Rv1261c | Novel | No |  |
| Rv1397c | VapC homolog, PIN domain | No |  |
| Rv1495 | MazF homolog | No |  |
| Rv1546 | Novel | No |  |
| Rv1561 | VapC homolog, PIN domain | Yes | Yes (Rv1560) |
| Rv1579c | Novel | No |  |
| Rv1583c | Novel | Not tested^a^ |  |
| Rv1720c | VapC homolog, PIN domain | No |  |
| Rv1741 | Novel | No |  |
| Rv1767 | Novel | No |  |
| **Protein** | **Method of identification** | **Toxic activity in *M. smegmatis*** | **TA system (antitoxin)** |
| Rv1838c | VapC homolog, PIN domain | No |  |
| Rv1942c | MazF homolog | Yes | Yes (Rv1943c) |
| Rv1953 | VapC homolog, PIN domain | No |  |
| Rv1955 | HigA homolog | Yes | Yes (Rv1956) |
| Rv1957 | Novel | No |  |
| Rv1959c | ParE homolog | No |  |
| Rv1962c | VapC homolog, PIN domain | Yes | Yes (Rv1962A) |
| Rv1982c | VapC homolog, PIN domain | No |  |
| Rv1991c | MazF homolog | Yes | Yes (Rv1991A) |
| Rv2010 | VapC homolog, PIN domain | Yes | Yes (Rv2009) |
| Rv2063A | MazF homolog | Yes | No (Rv2063) |
| Rv2103c | VapC homolog, PIN domain | Yes | Not tested |
| Rv2142c | ParE homolog | Not tested |  |
| Rv2231A | VapC homolog, PIN domain | No |  |
| Rv2274c | MazF homolog | Not tested^a^ |  |
| Rv2307B | Novel | Not tested^b^ |  |
| Rv2432c | Novel | Not tested |  |
| Rv2494 | VapC homolog, PIN domain | No |  |
| Rv2527 | VapC homolog, PIN domain | No |  |
| Rv2530c | VapC homolog, PIN domain | Yes | Yes (Rv2530A) |
| Rv2546 | VapC homolog, PIN domain | No |  |
| Rv2548 | VapC homolog, PIN domain | Yes | Yes (Rv2547) |
| Rv2549c | VapC homolog, PIN domain | No |  |
| Rv2562 | Novel | No |  |
| Rv2596 | VapC homolog, PIN domain | No |  |
| Rv2602 | VapC homolog, PIN domain | Yes | Yes (Rv2601A) |
| Rv2653c | Novel | Yes | Yes (Rv2654c) |
| Rv2656c | Novel | Not tested^a^ |  |
| Rv2705c | Novel | No |  |
| Rv2757c | VapC homolog, PIN domain | Yes | Yes (Rv2758c) |
| Rv2759c | VapC homolog, PIN domain | No |  |
| Rv2801c | MazF homolog | Yes | Yes (Rv2801A) |
| Rv2809 | Novel | No |  |
| Rv2829c | VapC homolog, PIN domain | Yes | Yes (Rv2830c) |
| Rv2863 | VapC homolog, PIN domain | No |  |
| Rv2866 | RelE homolog | Yes | Yes (Rv2865) |
| Rv2872 | VapC homolog, PIN domain | Yes | Yes (Rv2871) |
| Rv3180c | VapC homolog, PIN domain | Not tested |  |
| Rv3182 | Novel | No |  |
| **Protein** | **Method of identification** | **Toxic activity in *M. smegmatis*** | **TA system (antitoxin)** |
| Rv3224B | Novel | No |  |
| Rv3320c | VapC homolog, PIN domain | No |  |
| Rv3358 | RelE homolog | Yes | No (Rv3358) |
| Rv3384c | VapC homolog, PIN domain | Yes | Yes (Rv3385c) |
| Rv3408 | VapC homolog, PIN domain | Yes | Yes (Rv3407) |
| Rv3612c | Novel | No |  |
| Rv3655c | Novel | Not tested^b^ |  |
| Rv3697c | VapC homolog, PIN domain | Not tested |  |
| Rv3747 | Novel | No |  |
| Rv3705A | Novel | Not tested |  |

^a^ These proteins were found to be deleted in clinical isolates of *M. tuberculosis* H37Rv [11].

^b^ These proteins are different at the N or C terminus in *M. tuberculosis* H37Rv as compared to *M. tuberculosis* CDC1551.
