# Supplementary material for: Comprehensive Functional Analysis of Mycobacterium tuberculosis Toxin-Antitoxin Systems: Implications for Pathogenesis, Stress Responses, and Evolution
Source: PLoS Genet. 2009 Dec 11;5(12):e1000767. doi: 10.1371/journal.pgen.1000767 (PMC2781298; doi:10.1371/journal.pgen.1000767)
Supplement: Table S4 — Expression results for all genes tested during hypoxia. Results of qPCR for each M. tuberculosis gene tested in two experiments after induction of NRP (hypoxia). Data is expressed as gene/16S and the standard deviation (SD) at each timepoint is shown. Timepoints at which we were unable to detect signal for a given gene are indicated (ND). (0.12 MB DOC) [file pgen.1000767.s006.doc]

|  | **Hypoxia Experiment 1** | | | | | | | |
| --- | --- | --- | --- | --- | --- | --- | --- | --- |
| **Gene** | **Log** | **st. dev.** | **Day 2** | **st. dev.** | **Day 4** | **st. dev.** | **Day 10** | **st. dev.** |
| *hspX* | 0.025 | 0.005 | 0.167 | 0.0410 | 7.959 | 2.141 | 0.599 | 0.163 |
| *fdxA* | 0.093 | 0.012 | 0.915 | 0.1976 | 6.317 | 2.013 | 0.785 | 0.058 |
| *Rv0277A* | 1.925 | 0.305 | 1.953 | 0.6255 | 1.870 | 0.672 | 0.584 | 0.133 |
| *Rv0298* | 0.932 | 0.133 | 1.287 | 0.3801 | 2.481 | 0.904 | 0.693 | 0.156 |
| *Rv0300* | 1.424 | 0.080 | 1.577 | 0.5115 | 1.915 | 0.611 | 0.157 | 0.022 |
| *Rv0549c* | 0.822 | 0.194 | 3.766 | 0.6984 | 0.575 | 0.124 | 0.169 | 0.027 |
| *Rv0581* | 1.190 | 0.134 | 3.016 | 0.5234 | 0.525 | 0.117 | ND | ND |
| *Rv0608* | 1.585 | 0.275 | 2.046 | 0.4141 | 0.674 | 0.168 | 0.056 | 0.006 |
| *Rv0623* | 0.853 | 0.352 | 2.730 | 0.7417 | 1.485 | 0.559 | 0.380 | 0.044 |
| *Rv0909* | 1.741 | 0.142 | 0.957 | 0.5628 | 2.340 | 0.342 | 2.748 | 0.015 |
| *Rv1103c* | 0.728 | 0.046 | 2.917 | 0.6502 | 1.058 | 0.328 | 0.287 | 0.080 |
| *Rv1113* | 1.123 | 0.134 | 2.596 | 0.6448 | 1.700 | 0.518 | 0.478 | 0.024 |
| *Rv1241* | 1.551 | 0.110 | 2.540 | 0.3256 | 1.059 | 0.148 | 0.184 | 0.038 |
| *Rv1247c* | 1.085 | 0.124 | 1.691 | 0.5282 | 1.364 | 0.503 | 0.279 | 0.008 |
| *Rv1943c* | 1.108 | 0.223 | 2.688 | 0.6513 | 0.754 | 0.239 | 0.117 | 0.021 |
| *Rv1955* | 0.346 | 0.021 | 0.856 | 0.1064 | 1.926 | 0.311 | 3.023 | 0.467 |
| *Rv1991A* | 1.218 | 0.203 | 1.484 | 0.4042 | 1.946 | 0.386 | 0.364 | 0.070 |
| *Rv2009* | 0.423 | 0.021 | 3.482 | 0.5803 | 0.294 | 0.066 | 0.135 | 0.017 |
| *Rv2103c* | 1.017 | 0.094 | 2.211 | 0.4823 | 1.008 | 0.257 | 0.135 | 0.022 |
| *Rv2530A* | 1.930 | 0.189 | 2.573 | 0.5426 | 1.764 | 0.539 | 0.322 | 0.029 |
| *Rv2547* | 0.836 | 0.136 | 2.942 | 1.0544 | 0.784 | 0.245 | 0.333 | 0.066 |
| *Rv2653c* | 0.394 | 0.050 | 2.871 | 0.7871 | 1.099 | 0.440 | 1.042 | 0.352 |
| *Rv2758c* | 1.230 | 0.474 | 2.709 | 0.9300 | 0.199 | 0.072 | 0.065 | 0.017 |
| *Rv2829c* | 2.435 | 0.432 | 3.198 | 0.4035 | 1.868 | 0.399 | 0.756 | 0.092 |
| *Rv2865* | 3.389 | 0.320 | 2.354 | 0.4374 | 0.840 | 0.187 | 0.368 | 0.033 |
| *Rv2872* | 1.505 | 0.290 | 2.197 | 0.3771 | 0.586 | 0.107 | 0.290 | 0.053 |
| *Rv3407* | 1.442 | 0.248 | 1.853 | 0.4293 | 1.864 | 0.377 | 0.444 | 0.095 |

|  | **Hypoxia Experiment 2** | | | | | | | |
| --- | --- | --- | --- | --- | --- | --- | --- | --- |
| **Gene** | **Log** | **st. dev.** | **Day 2** | **st. dev.** | **Day 4** | **st. dev.** | **Day 10** | **st. dev.** |
| *hspX* | 0.005 | 0.001 | 2.695 | 0.355 | 1.580 | 0.322 | 0.801 | 0.066 |
| *fdxA* | 0.011 | 0.027 | 4.947 | 0.617 | 1.045 | 0.146 | 0.581 | 0.034 |
| *Rv0277A* | 0.816 | 0.091 | 1.226 | 0.345 | 1.015 | 0.242 | 1.259 | 0.328 |
| *Rv0298* | 0.889 | 0.080 | 0.543 | 0.069 | 0.669 | 0.163 | 1.349 | 0.168 |
| *Rv0300* | 0.900 | 0.007 | 1.736 | 0.024 | 0.478 | 0.039 | 0.667 | 0.055 |
| *Rv0549c* | 1.138 | 0.161 | 2.506 | 0.305 | 1.455 | 0.384 | 1.800 | 0.341 |
| *Rv0581* | 1.199 | 0.105 | 0.954 | 0.232 | 1.019 | 0.255 | 0.854 | 0.135 |
| *Rv0608* | 1.199 | 0.237 | 1.670 | 0.317 | 1.056 | 0.228 | 0.610 | 0.155 |
| *Rv0623* | 1.203 | 0.167 | 1.213 | 0.149 | 1.534 | 0.178 | 1.940 | 0.799 |
| *Rv0909* | 1.775 | 0.150 | 1.787 | 0.526 | 0.362 | 0.078 | 0.578 | 0.127 |
| *Rv1103c* | 1.587 | 0.320 | 0.816 | 0.137 | 0.344 | 0.068 | 0.914 | 0.197 |
| *Rv1113* | 1.671 | 0.176 | 1.955 | 0.291 | 0.311 | 0.077 | 0.744 | 0.148 |
| *Rv1241* | 1.001 | 0.086 | 3.258 | 0.459 | 0.808 | 0.150 | 0.700 | 0.185 |
| *Rv1247c* | 1.237 | 0.198 | 2.194 | 0.312 | 0.570 | 0.069 | 1.268 | 0.280 |
| *Rv1943c* | 1.262 | 0.177 | 3.072 | 0.645 | 0.255 | 0.053 | 0.781 | 0.096 |
| *Rv1955* | 0.379 | 0.047 | 0.893 | 0.140 | 4.089 | 0.907 | 1.322 | 0.793 |
| *Rv1991A* | 0.504 | 0.043 | 2.688 | 0.737 | 0.998 | 0.225 | 1.963 | 0.351 |
| *Rv2009* | 0.297 | 0.044 | 3.706 | 0.520 | 1.510 | 0.269 | 3.360 | 0.741 |
| *Rv2103c* | 1.111 | 0.088 | 2.662 | 0.739 | 0.498 | 0.124 | 1.276 | 0.381 |
| *Rv2530A* | 1.427 | 0.113 | 2.385 | 0.583 | 0.547 | 0.118 | 1.610 | 0.418 |
| *Rv2547* | 0.772 | 0.081 | 1.641 | 0.318 | 0.269 | 0.072 | 0.833 | 0.111 |
| *Rv2653c* | 1.084 | 0.188 | 2.576 | 0.414 | 0.557 | 0.081 | 2.859 | 0.412 |
| *Rv2758c* | 1.437 | 0.232 | 2.308 | 0.300 | 0.434 | 0.050 | 1.443 | 0.247 |
| *Rv2829c* | 0.745 | 0.066 | 3.581 | 0.532 | 1.464 | 0.236 | 0.693 | 0.141 |
| *Rv2865* | 1.752 | 0.223 | 1.331 | 0.288 | 1.592 | 0.407 | 2.045 | 0.508 |
| *Rv2872* | 1.749 | 0.139 | 1.273 | 0.271 | 0.520 | 0.103 | 1.526 | 0.188 |
| *Rv3407* | 1.003 | 0.132 | 1.716 | 0.304 | 1.330 | 0.312 | 3.328 | 0.492 |
