# Supplementary material for: Comprehensive Functional Analysis of Mycobacterium tuberculosis Toxin-Antitoxin Systems: Implications for Pathogenesis, Stress Responses, and Evolution
Source: PLoS Genet. 2009 Dec 11;5(12):e1000767. doi: 10.1371/journal.pgen.1000767 (PMC2781298; doi:10.1371/journal.pgen.1000767)
Supplement: Table S5 — Expression results for all genes tested during macrophage infection. Results of qPCR for each M. tuberculosis gene tested in two experiments at 4 and 24 h after infection of IFN-γ-stimulated wild-type macrophages. Data is expressed as gene/16S and the standard deviation (SD) at each timepoint is shown. (0.13 MB DOC) [file pgen.1000767.s007.doc]

|  | **Macrophage Infection 1** | | | | | |
| --- | --- | --- | --- | --- | --- | --- |
| **Gene** | **Log** | **st. dev.** | **4 h** | **st. dev** | **24 h** | **st. dev.** |
| *hspX* | 0.004 | 0.000 | 1.019 | 0.130 | 3.137 | 0.317 |
| *icl* | 0.008 | 0.001 | 2.746 | 0.456 | 1.618 | 0.365 |
| *fdxA* | 0.035 | 0.006 | 1.556 | 0.283 | 1.653 | 0.445 |
| *Rv0277A* | 1.997 | 0.252 | 1.619 | 0.252 | 0.529 | 0.088 |
| *Rv0298* | 2.406 | 0.245 | 1.114 | 0.138 | 0.727 | 0.081 |
| *Rv0300* | 2.045 | 0.179 | 1.530 | 0.228 | 0.770 | 0.163 |
| *Rv0549c* | 1.015 | 0.120 | 2.378 | 0.357 | 0.954 | 0.155 |
| *Rv0608* | 4.248 | 0.487 | 2.200 | 0.386 | 0.840 | 0.175 |
| *Rv0623* | 2.687 | 0.363 | 1.172 | 0.241 | 0.647 | 0.052 |
| *Rv0909* | 2.636 | 0.168 | 1.236 | 0.171 | 0.262 | 0.042 |
| *Rv1103c* | 2.392 | 0.168 | 1.181 | 0.180 | 0.245 | 0.044 |
| *Rv1113* | 2.359 | 0.226 | 0.980 | 0.245 | 0.143 | 0.015 |
| *Rv1241* | 1.553 | 0.058 | 2.315 | 0.468 | 0.753 | 0.100 |
| *Rv1247c* | 1.956 | 0.220 | 2.468 | 0.351 | 0.775 | 0.172 |
| *Rv1560* | 0.565 | 0.060 | 2.185 | 0.261 | 1.560 | 0.294 |
| *Rv1955* | 1.016 | 0.121 | 0.583 | 0.034 | 1.940 | 0.318 |
| *Rv1991A* | 1.120 | 0.160 | 1.480 | 0.142 | 0.874 | 0.156 |
| *Rv2009* | 1.387 | 0.106 | 1.860 | 0.268 | 0.623 | 0.131 |
| *Rv2103c* | 1.633 | 0.046 | 1.958 | 0.348 | 0.847 | 0.112 |
| *Rv2530A* | 2.497 | 0.270 | 1.526 | 0.245 | 0.492 | 0.089 |
| *Rv2547* | 1.943 | 0.205 | 1.324 | 0.194 | 0.622 | 0.118 |
| *Rv2653c* | 1.897 | 0.336 | 3.928 | 0.587 | 0.758 | 0.212 |
| *Rv2758c* | 2.320 | 0.417 | 1.309 | 0.194 | 0.556 | 0.146 |
| *Rv2829c* | 1.464 | 0.081 | 1.394 | 0.203 | 0.533 | 0.148 |
| *Rv2865* | 3.760 | 0.463 | 1.464 | 0.217 | 0.451 | 0.124 |
| *Rv3407* | 2.366 | 0.166 | 1.412 | 0.205 | 0.624 | 0.103 |

|  | **Macrophage Infection 2** | | | | | |
| --- | --- | --- | --- | --- | --- | --- |
| **Gene** | **Log** | **st. dev.** | **4 h** | **st. dev** | **24 h** | **st. dev.** |
| *hspX* | 0.003 | 0.001 | 0.205 | 0.049 | 2.071 | 0.497 |
| *icl* | 0.041 | 0.003 | 1.886 | 0.091 | 1.230 | 0.218 |
| *fdxA* | 0.019 | 0.002 | 0.756 | 0.136 | 1.959 | 0.119 |
| *Rv0277A* | 2.707 | 0.202 | 1.301 | 0.129 | 1.058 | 0.166 |
| *Rv0298* | 5.685 | 0.808 | 1.014 | 0.343 | 0.685 | 0.142 |
| *Rv0300* | 2.228 | 0.160 | 1.324 | 0.106 | 1.060 | 0.162 |
| *Rv0549c* | 0.927 | 0.104 | 3.143 | 0.867 | 2.225 | 0.417 |
| *Rv0608* | 3.385 | 0.207 | 1.721 | 0.168 | 1.994 | 0.368 |
| *Rv0623* | 7.219 | 0.546 | 0.731 | 0.131 | 0.821 | 0.123 |
| *Rv0909* | 5.198 | 1.198 | 1.039 | 0.156 | 0.621 | 0.127 |
| *Rv1103c* | 3.306 | 0.521 | 0.984 | 0.199 | 0.559 | 0.084 |
| *Rv1113* | 3.566 | 0.881 | 0.990 | 0.128 | 0.612 | 0.086 |
| *Rv1241* | 2.931 | 0.575 | 1.735 | 0.157 | 0.833 | 0.164 |
| *Rv1247c* | 2.486 | 0.749 | 1.624 | 0.285 | 1.104 | 0.274 |
| *Rv1560* | 0.881 | 0.342 | 2.020 | 0.474 | 2.222 | 0.459 |
| *Rv1955* | 1.100 | 0.128 | 0.682 | 0.095 | 1.602 | 0.213 |
| *Rv1991A* | 1.280 | 0.216 | 1.036 | 0.156 | 1.303 | 0.183 |
| *Rv2009* | 2.948 | 0.300 | 1.849 | 0.404 | 1.232 | 0.207 |
| *Rv2103c* | 2.780 | 0.313 | 1.851 | 0.360 | 1.226 | 0.304 |
| *Rv2530A* | 3.183 | 0.261 | 1.217 | 0.055 | 0.964 | 0.146 |
| *Rv2547* | 4.572 | 0.305 | 1.135 | 0.070 | 1.378 | 0.190 |
| *Rv2653c* | 1.785 | 0.322 | 1.731 | 0.281 | 1.429 | 0.224 |
| *Rv2758c* | 4.790 | 0.572 | 0.994 | 0.156 | 0.599 | 0.052 |
| *Rv2829c* | 1.304 | 0.154 | 1.312 | 0.274 | 1.350 | 0.182 |
| *Rv2865* | 4.112 | 1.168 | 1.382 | 0.189 | 1.334 | 0.289 |
| *Rv3407* | 1.753 | 0.168 | 1.001 | 0.147 | 0.702 | 0.112 |
