# Supplementary material for: Comprehensive Functional Analysis of Mycobacterium tuberculosis Toxin-Antitoxin Systems: Implications for Pathogenesis, Stress Responses, and Evolution
Source: PLoS Genet. 2009 Dec 11;5(12):e1000767. doi: 10.1371/journal.pgen.1000767 (PMC2781298; doi:10.1371/journal.pgen.1000767)
Supplement: Table S6 — Vectors, plasmids, and primers used in this study. (0.20 MB DOC) [file pgen.1000767.s008.doc]

| **Strains** | **Description** | **Reference** |  |  |
| --- | --- | --- | --- | --- |
| mc^2^155 | *M. smegmatis* laboratory strain | 1 |  |  |
| Erdman | virulent *M. tuberculosis* strain | W. R. Jacobs, Jr. |  |  |
| DH5α | *E.coli;* Fˉ Φ80*lac*ZΔM15 Δ(*lac*ZYA-*arg*F)U169 *rec*A1 *end*A1 *hsd*R17(r_k_ˉm_k_^+^) *pho*A *sup*E44 *thi*-1 *gyr*A96 *rel*A1 λˉ | Invitrogen |  |  |
| BL21(DE3) pLysS | *E. coli*; Fˉ *ompT* *hsdS*_B_ (r_B_ˉm_B_ˉ) *gal dcm* (DE3) pLysS (Cam^R^) | Invitrogen |  |  |
|  |  |  |  |  |
| **Vector** | **Description** | **Reference** | **Forward primer** | **Reverse Primer** |
| pMV261.kan | *oriE,* *oriM*, Kan^R^ | W. R. Jacobs, Jr. |  |  |
| pHR100 | pMV261.kan+ acetamidase promoter | This study, 3 |  |  |
| pHR101 | pHR100 + *Rv0065* | This study | TGTGGATGAATGTGTAGTCGACG | CGCGGATCCTCACCGAACGAGTTTGATTTCG |
| pHR102 | pHR100 + *Rv0298* | This study | GACGAAAGAGAAGATCTCCGTG | CGCGGATCCTCAACTTCCGGCCGCCCGG |
| pHR103 | pHR100 + *Rv1246c* | This study | TGTGAGCGACGACCATCCCTA | CGCGGATCCTTAACGTGGCCGGCACGGGTT |
| pHR104 | pHR100 + *Rv1955* | This study | TGTGCCGTCGGGATGGGTGT | CGCGGATCCTCAGATCGGTGGGGTGTCGC |
| pHR105 | pHR100 + *Rv1991c* | This study | TGTGGTGATTAGTCGTGCCGAG | CGCGGATCCTCAAAGGTCCAGTACGCGACG |
| pHR106 | pHR100 + *Rv2801c* | This study | TGTGATGCGCCGCGGTGAGAT | CGCGGATCCCTACGACCATAAGTCGAGATG |
| pHR107 | pHR100 + *Rv2866* | This study | TGTGCCTTACACCGTGCGGTT | CGCGGATCCCTATCGGCGGTAGATGTCCG |
| pHR108 | pHR100 + *Rv0301* | This study | TGTGACTGACCAGCGCTGGCT | CGCGGATCCTTAAGCGGAAGGCGGGCGAT |
| pHR109 | pHR100 + *Rv0456A* | This study | GCTGCGCGGTGAGATCTGG | CGCGGATCCTCAGCTCGGCAGGGGAGAC |
| pHR110 | pHR100 + *Rv0549c* | This study | TGTGAGAGCATCGCCCACTTC | CGCGGATCCTCAGCCGATGGCGTGAGCC |
| pHR111 | pHR100 + *Rv0595c* | This study | TGTGAACGTACGGCGCGCCC | CGCGGATCCTCAGATCGTTATGACCTCGACG |
| pHR112 | pHR100 + *Rv0598c* | This study | TGTGAAACCGCCGCTGGCAGT | CGCGGATCCTCACGCGGCGACAACGACGT |
| pHR113 | pHR100 + *Rv0627* | This study | TGTGAGCACGACGCCGGCC | CGCGGATCCTCAGACCCGAATGATCTCCAC |
| pHR114 | pHR100 + *Rv0656c* | This study | TTGGCGGCTGCAACGACAAC | CGCGGATCCCTAGTCGTCGGCGCTGACGA |
| pHR115 | pHR100 + *Rv0659c* | This study | GCGGCGCGGTGAATTGTGGT | CGCGGATCCTCAACACCCCGTGCTCGCC |
| pHR116 | pHR100 + *Rv0661c* | This study | GATAGTGCTCGACACCACCG | CGCGGATCCTCAGCGGTCGCCGAGCAGG |
| pHR117 | pHR100 + *Rv0665* | This study | TGTGACCGAGGGCGAGGTCG | CGCGGATCCTCACACCTTGATCTCCGCGG |
| **Vector** | **Description** | **Reference** | **Forward primer** | **Reverse Primer** |
| pHR118 | pHR100 + *Rv0960* | This study | GATCGTCGTTGACGCCTCGG | CGCGGATCCCTACCTGGGCACAACGGTAAT |
| pHR119 | pHR100 + *Rv1102c* | This study | GCGACCTATCCACATCGCAC | CGCGGATCCCTATGCCACCACCCAATCGAG |
| pHR120 | pHR100 + *Rv0277c* | This study | GTTCCTCATCGACGTCAACG | CGCGGATCCCTAGATTGGCGGGCGAATAT |
| pHR121 | pHR100 + *Rv1397c* | This study | GATCCTTGTCGACTCCGATGT | CGCGGATCCTCATGCCCGCGGTCGGTGC |
| pHR122 | pHR100 + *Rv1495* | This study | TGTGAACGCGCCGTTGCGTG | CGCGGATCCTCATGGCCACGGTAGCCCCA |
| pHR123 | pHR100 + *Rv1561* | This study | GATCCTCATCGACACATCGGC | CGCGGATCCTCACCGAAACGATGCGGCCT |
| pHR124 | pHR100 + *Rv1720c* | This study | TGTGATCGTGTTGGACGCCTC | CGCGGATCCTCAGGCGACAAGCTCGATCTC |
| pHR125 | pHR100 + *Rv0543c* | This study | TGTGAACCGGTTTCTCACCTC | CGCGGATCCCTATGCGTGCTCCTCGCGGT |
| pHR126 | pHR100 + *Rv0582* | This study | TGTGATCATCGACACGAGTGC | CGCGGATCCTTACGGAATGACGGTGAAGC |
| pHR127 | pHR100 + *Rv0609* | This study | TGTGATCGTCGACACGTCGG | CGCGGATCCTCACCGCCGATCCAGTGC |
| pHR128 | pHR100 + *Rv0617* | This study | TGTGACGGTGCTGCTCGACGC | CGCGGATCCTCAGGTGGTCGTTGGAATGA |
| pHR129 | pHR100 + *Rv0624* | This study | GGTGATCGACACGTCCGCG | CGCGGATCCTTAGGGCAGCGCGACCGTGG |
| pHR130 | pHR100 + *Rv0749* | This study | GTTCCTTCTCGACGCCAACG | CGCGGATCCCTAGAGCGGCGGGCGAATGT |
| pHR131 | pHR100 + *Rv0948c* | This study | GAGACCAGAACCCCCACATC | CGCGGATCCTTAGTGACCGAGGCGGCCCC |
| pHR132 | pHR100 + *Rv1114* | This study | TGTGATCCTGGTCGACACTTC | CGCGGATCCTCAGGACACTTCCTCAGCCA |
| pHR133 | pHR100 + *Rv1242* | This study | TGTGATCATCCCTGACATCAATC | CGCGGATCCTTATTCGCGCAACGGGTCGG |
| pHR134 | pHR100 + *Rv1741* | This study | GGTGATCGATACCTCTGCGC | CGCGGATCCTCAGCCGGCGAGAACCGCG |
| pHR135 | pHR100 + *Rv1838c* | This study | TGTGATCCTCGTTGACTCCAAC | CGCGGATCCTCAGGCCAGACGCTTGATCC |
| pHR136 | pHR100 + *Rv1942c* | This study | TGTGACCGCACTTCCGGCGC | CGCGGATCCTCATCGAGAGCAATCGACGGC |
| pHR137 | pHR100 + *Rv1953* | This study | TGTGACCTACGTCCTGGACAC | CGCGGATCCCTAAAACCAGCTTATACCAG |
| pHR138 | pHR100 + *Rv1957* | This study | GACTGACCGAACCGACGCC | CGCGGATCCTCAGGGCGTTCCTCTCGTTG |
| pHR139 | pHR100 + *Rv1959c* | This study | TGTGAGTAGCCGATACCTTCTCT | CGCGGATCCTCAGAGGTTCCGGTCGACGT |
| pHR140 | pHR100 + *Rv2010* | This study | GATCGTCGACACCTCGGTCT | CGCGGATCCTCAGAACAACGGCTCGGTGC |
| pHR141 | pHR100 + *Rv2527* | This study | GACCACCTGGATTCTGGACAA | CGCGGATCCCTAGCGCCCGCGAGAGAGTT |
| pHR142 | pHR100 + *Rv2546* | This study | GGTGTTCTGCGTCGACACCA | CGCGGATCCTTAAAGGGTCCCGCGCGGC |
| pHR143 | pHR100 + *Rv2548* | This study | TGTGAAGCTGATCGACACCACC | CGCGGATCCTCAGTACGGCGGCTGCAGAT |
| pHR144 | pHR100 + *Rv2549c* | This study | GATCTTCGTCGACACGTCCTT | CGCGGATCCCTACTCGGGTCTTACCTCGAC |
| pHR145 | pHR100 + *Rv2596* | This study | TGTGATCGCACCAGACACCAG | CGCGGATCCTCAGGTCACCAGCTCAACCTC |
| pHR146 | pHR100 + *Rv2602* | This study | GCTGCTCTGTGACACCAACAT | CGCGGATCCTCACTTGGCCAGGAGCCGCA |
| pHR147 | pHR100 + *Rv2757c* | This study | GACCACGCGCTATTTGCTCG | CGCGGATCCTCACGCTGTCCCTGGGGCC |
| pHR148 | pHR100 + *Rv2829c* | This study | GACGACGGTGCTGCTCGACT | CGCGGATCCCTACCAGACGGTGACCGGTC |
| **Vector** | **Description** | **Reference** | **Forward primer** | **Reverse Primer** |
| pHR149 | pHR100 + *Rv2863* | This study | GATCTTCGTCGATACCAACGTC | CGCGGATCCTCATGATCGGAATGCGCTGGC |
| pHR150 | pHR100 + *Rv3358* | This study | TGTGAGAAGCGTCAACTTCGATC | CGCGGATCCTCAGTAGTGGTATCGGGCCTT |
| pHR151 | pHR100 + *Rv3384c* | This study | GGCGGCCATCTATCTCGACT | CGCGGATCCTCACGACGGGGCGATCACG |
| pHR152 | pHR100 + *Rv3408* | This study | TGTGATCTATATGGACACCTCGG | CGCGGATCCTCACCGGACTGCGCCGGGT |
| pHR153 | pHR100 + *Rv2063A* | This study | TTGGCTGAGCCACGGCGAG | CGCGGATCCTCACGGGTCCCGGCCAC |
| pHR154 | pHR100 + *Rv0240* | This study | TGTGCTCTCGATCGATACGAA | CGCGGATCCTCAGCCATCCGACGTTATCG |
| pHR155 | pHR100 + *Rv0299* | This study | TTGATCGCTCCCGGCGACAT | CGCGGATCCCTAACAGAGCAGAGCTGTCA |
| pHR156 | pHR100 + *Rv0910* | This study | GGCGAAACTGTCCGGATCCA | CGCGGATCCTCAGCCGGCAAACACCGTGA |
| pHR158 | pHR100 + *Rv1261c* | This study | GGACATTTCCCGATGGCTTG | CGCGGATCCTTAGCGGCGGGTCAATACGA |
| pHR159 | pHR100 *+ Rv1579c* | This study | TGTGACCCCGATCAACCGGCC | CGCGGATCCTCACGATGGCGACCCC |
| pHR160 | pHR100 + *Rv1767* | This study | GTCGGACCAGCCACGTCATC | CGCGGATCCCTAGGACGGCGTTGTGTCA |
| pHR161 | pHR100 + *Rv1982c* | This study | GATCGTGGACACAAGCGCCG | CGCGGATCCTTACGCGACGCCTGGCCAGT |
| pHR162 | pHR100 + *Rv2530c* | This study | TGTGACGGCACTGCTCGATGT | CGCGGATCCCTACAACACCTCGACGAGGC |
| pHR163 | pHR100 + *Rv2809* | This study | GACGTACGCAGCCAGGGACG | CGCGGATCCTCATCGGTTCCACTCCACCA |
| pHR164 | pHR100 + *Rv2653c* | This study | TTGACCCACAAGCGCACTAA | CGCGGATCCTCACTGTTTGCTGTCGGGTT |
| pHR165 | pHR100 + *Rv2872* | This study | GCTGTGCGTTGATGTCAACG | CGCGGATCCTTATAGATGGGTCTGACCGT |
| pHR166 | pHR100 + *Rv3183* | This study | GACCATGGCCCGCAACTGGC | CGCGGATCCTCAGGCGGTCAGCTCGACAG |
| pHR167 | pHR100 + *Rv3747* | This study | TGTGATACTTACGGGTGCGTT | CGCGGATCCTCAAAAACCTATCGTCGCAG |
| pHR168 | pHR100 *+ Rv2562* | This study | TGTGGCCGAACAAAAGGTGAA | CGCGGATCCTTAGCCCATTTCGGCGGGCT |
| pHR169 | pHR100 + *Rv1560-1561* | This study | TGTGTATCGTTGGTGTATGTCG | CGCGGATCCTCACCGAAACGATGCGGCCT |
| pHR170 | pHR100 + *Rv0300-0301* | This study | GAGTGATGTACTGATTCGGGAC | CGCGGATCCTTAAGCGGAAGGCGGGCGAT |
| pHR171 | pHR100 + *Rv3357-3358* | This study | GAGCATCAGTGCGAGCGAGG | CGCGGATCCTCAGTAGTGGTATCGGGCCTT |
| pHR172 | pHR100 + *Rv0549c-0550c* | This study | TTGTTGAGCCGGCGCACGAA | CGCGGATCCTCAGCCGATGGCGTGAGCC |
| pHR173 | pHR100 + *Rv2829c-2830c* | This study | GACCGCTACGGAGGTGAAG | CGCGGATCCCTACCAGACGGTGACCGGTC |
| pHR174 | pHR100 + *Rv3407-3408* | This study | GCGTGCTACCGTTGGGCTTGT | CGCGGATCCTCACCGGACTGCGCCGGGT |
| pHR175 | pHR100 + *Rv2705c* | This study | TGTGAGAATGACGCCCGATCC | CGCGGATCCCTACGTAGGGTCGGCGGTTG |
| pHR176 | pHR100 + *Rv0760c* | This study | GACCCAAACGACCCAATCCC | CGCGGATCCTCATTCCTGGTTACCGAAGG |
| pHR177 | pHR100 + *Rv2759c* | This study | TGTGATCGTCGATACGTCGGC | CGCGGATCCTCAGGTGCACGCGGGCCGGA |
| pHR178 | pHR100 + *Rv2757c-2758c* | This study | GCATCGCGGATATGCATTAGTA | CGCGGATCCTCACGCTGTCCCTGGGGCC |
| pHR179 | pHR100 + *Rv2601A-2602* | This study | GAAGACCACGCTCGACCTGC | CGCGGATCCTCACTTGGCCAGGAGCCGCA |
| pHR180 | pHR100 + *Rv2231A* | This study | GGCGTGTACCGCGTGCCC | CGCGGATCCTCACAGGCAGCTAACAGGGC |
| **Vector** | **Description** | **Reference** | **Forward primer** | **Reverse Primer** |
| pHR181 | pHR100 + *Rv2494* | This study | TGTGGCGCTGCTCGACGTCAA | TTAGAGGATGGTCAGCAGCT |
| pHR182 | pHR100 *+ Rv3224B* | This study | TGTGCCCAAAGCGGCCATGGC | CGCGGATCCTTAGCGATGATCGCGCTGAT |
| pHR183 | pHR100 + *Rv1102c-1103c* | This study | GTACCTACCCTGGGGGGTC | CGCGGATCCCTATGCCACCACCCAATCGAG |
| pHR184 | pHR100 + *Rv0623-0624* | This study | ATGGCGCTGAGTATCAAGCA | CGCGGATCCTTAGGGCAGCGCGACCGTGG |
| pHR185 | pHR100 + *Rv0477* | This study | GAAAGCCCTGGTGGCCGTGT | CGCGGATCCCTAAAACGACTCTTCAGGGC |
| pHR186 | pHR100 + *Rv0909-0910* | This study | ATGGGAATCCTGGACAAGGT | CGCGGATCCTCAGCCGGCAAACACCGTGA |
| pHR187 | pHR100 + *Rv1962c* | This study | TGTGATCTACCTGGAAACCTC | CGCGGATCCTCATCGCGCTTGTCCGGGT |
| pHR188 | pHR100 + *Rv2063-2063A* | This study | ATGTCTACATCCACGACGAT | CGCGGATCCTCACGGGTCCCGGCCAC |
| pHR189 | pHR100 + *Rv1942c-1943c* | This study | TGTGAAGACGGCCCGGTTGCA | CGCGGATCCTCATCGAGAGCAATCGACGGC |
| pHR190 | pHR100 + *Rv2530A-2530c* | This study | ATGCGCACCACGTTGCAGAT | CGCGGATCCCTACAACACCTCGACGAGGC |
| pHR191 | pHR100 + *Rv2103c* | This study | GAAGATCGTCGACGCGAACG | CGCGGATCCTCACAACAGCGCGGGCG |
| pHR192 | pHR100 + *Rv2009-2010* | This study | TGTGTATAGTGGTGTTGTGTCAC | CGCGGATCCTCAGAACAACGGCTCGGTGC |
| pHR193 | pHR100 + *Rv1241-1242* | This study | ATGCGCACCACCTTGACGCT | CGCGGATCCTTATTCGCGCAACGGGTCGG |
| pHR194 | pHR100 + *Rv3182* | This study | TTGGCCGTGATCCTGCTCCC | TCATCCACCTCCGTGCTCGC |
| pHR195 | pHR100 + *Rv2865-2866* | This study | GCGGATACTGCCGATTTCGAC | CGCGGATCCCTATCGGCGGTAGATGTCCG |
| pHR196 | pHR100 + *Rv1246c-1247c* | This study | GGCTGTTGTCCCACTGGGC | CGCGGATCCTTAACGTGGCCGGCACGGGTT |
| pHR197 | pHR100 + *Rv0277A-0277c* | This study | GTGATCGAAGACGCCCTTCG | CGCGGATCCCTAGATTGGCGGGCGAATAT |
| pHR198 | pHR100 + *Rv2871-2872* | This study | TGTGCGCACGACGATCCGTAT | CGCGGATCCTTATAGATGGGTCTGACCGT |
| pHR199 | pHR100 + *Rv0748-0749* | This study | ATGCGCACCACGGTGTCAAT | CGCGGATCCCTAGAGCGGCGGGCGAATGT |
| pHR200 | pHR100 + *Rv0298-0299* | This study | GACGAAAGAGAAGATCTCCGTG | CGCGGATCCCTAACAGAGCAGAGCTGTCA |
| pHR201 | pHR100 + *Rv0581-0582* | This study | GTGGACAAGACGACGGTCTA | CGCGGATCCTTACGGAATGACGGTGAAGC |
| pHR202 | pHR100 + *Rv3320c* | This study | GCGAGCGCTGTTGGACGTCA | CGCGGATCCCTACAGCGTTGCCAGGTGCT |
| pHR203 | pHR100 + *Rv1113-1114* | This study | ATGAGGACGACGGTGACCGT | CGCGGATCCTCAGGACACTTCCTCAGCCA |
| pHR204 | pHR100 + *Rv1546* | This study | GGCCTCTGTTGAGCTGTCCG | CTAGCCGTATAGCTCGGCGA |
| pHR205 | pHR100 + *Rv1962A-1962c* | This study | TGTGAATGAGGTGTCCATACG | CGCGGATCCTCATCGCGCTTGTCCGGGT |
| pHR206 | pHR100 + *Rv2547-2548* | This study | GCGGACTCAGGTGACCCTG | CGCGGATCCTCAGTACGGCGGCTGCAGAT |
| pHR207 | pHR100 + *Rv2653c-2654c* | This study | GAGCGGCCACGCGTTGGC | CGCGGATCCTCACTGTTTGCTGTCGGGTT |
| pHR208 | pHR100 + *Rv2801A-2801c* | This study | TGTGAAGTTGAGCGTGAGCCTG | CGCGGATCCCTACGACCATAAGTCGAGATG |
| pHR209 | pHR100 + *Rv3384c-Rv3385c* | This study | GACGCCGACCGCTTGTGCT | CGCGGATCCTCACGACGGGGCGATCACG |
| pHR210 | pHR100 + *Rv3612c* | This study | GGTCGCTGTGCTCACCTATG | CGCGGATCCTCACCCCAGAGCCGGGTGCA |
| pHR211 | pHR100 + *Rv0608-0609* | This study | GGCGTTGAACATCAAAGA | CGCGGATCCTCACCGCCGATCCAGTGC |
| **Vector** | **Description** | **Reference** | **Forward primer** | **Reverse Primer** |
| pLIC-HMK | Ligation independent cloning vector for protein expression | Gift of Dr. James Berger |  |  |
| pHR212 | pLIC-HMK + *mazF* | This study | TACTTCCAATCCAATGCAATGGTAAGCCGATACGTACC | TTATCCACTTCCAATGTTATTACTACCCAATCAGTACGTTAA |
| pHR214 | pLIC-HMK + *Rv0301* | This study | TACTTCCAATCCAATGCAGTGACTGACCAGCGCTGG | TTATCCACTTCCAATGTTATTATTAAGCGGAAGGCGGGCG |
| pHR215 | pLIC-HMK + *Rv0910* | This study | TACTTCCAATCCAATGCAATGGCGAAACTGTCCGGATC | TTATCCACTTCCAATGTTATTATCAGCCGGCAAACACCGTGA |
| pHR216 | pLIC-HMK + *R1561* | This study | TACTTCCAATCCAATGCAATGATCCTCATCGACACA | TTATCCACTTCCAATGTTATTATCACCGAAACGATGCGGC |
| pHR217 | pLIC-HMK + *Rv2829c* | This study | TACTTCCAATCCAATGCAATGACGACGGTGCTGCTC | TTATCCACTTCCAATGTTATTACTACCAGACGGTGACCGG |
| pHR218 | pLIC-HMK + *Rv2866* | This study | TACTTCCAATCCAATGCAGTGCCTTACACCGTGCGG | TTATCCACTTCCAATGTTATTACTATCGGCGGTAGATGTC |
| pUV15tetORm | Tetracycline-inducible expression vector | 2 |  |  |
| pLC436 | pUV15tetORm + *MSMEG_5634* | This study | gcttaattaagaaggagatatacatatggccaaactttccgtctc | aactgcagaggtgctcaagcgtgaactc |
| pLC437 | pUV15tetORm + *MSMEG_5634-5635* | This study | gcttaattaagaaggagatatacatatgggattcctggacaaggc | aactgcagaggtgctcaagcgtgaactc |
