# Supplementary material for: Comprehensive Functional Analysis of Mycobacterium tuberculosis Toxin-Antitoxin Systems: Implications for Pathogenesis, Stress Responses, and Evolution
Source: PLoS Genet. 2009 Dec 11;5(12):e1000767. doi: 10.1371/journal.pgen.1000767 (PMC2781298; doi:10.1371/journal.pgen.1000767)
Supplement: Table S7 — Primers used for qPCR of toxin, antitoxin, and control genes. (0.05 MB DOC) [file pgen.1000767.s009.doc]

| **Gene** | **Forward primer** | **Reverse primer** |
| --- | --- | --- |
| *16S* | ATGCTACAATCGCCGGTACA | GCGTTGCTGATCTGCGAT |
| *hspX* | CCGAGCGCACCGAGCAGAAG | GGTGGCCTTAATGTCGTCCTCGTC |
| *fdxA* | CCGCGTCGAGGCGATCTACT | CGACCAGAGGCGTGTCGACT |
| *icl* | CCATGGGCTTCAAGTTCCAGTTCA | TTCGACATACGCGCTCATCTGGTT |
| *recA* | AGAAGATCGGTGTGATGTTCGGCT | ACCTTGTTCTTGACGACCTTGACG |
| *Rv0277A* | GGCCAGGTATCGACCTAACCTCGAAT | TCACTTCCGCGAGTTCAGTTCCA |
| *Rv0298* | GACGAAAGAGAAGATCTCCGTGAC | AGTGCCTGCTCAATCATTTCCGAC |
| *Rv0300* | ACGACGTGTTAGCAAGCCTTGACG | GGCTAAACGCCGACGGATGTATTC |
| *Rv0549c* | ACTCACCGTTGCCTATGTCGAT | TCTTTCGTCGGTGGTCAACAACAC |
| *Rv0581* | GGTCTACCTGCCGGATGAACTCAA | GGATTACCTGCGCTTCGGAGACT |
| *Rv0608* | AAATCACCGGCGAATCTCAGGCT | ATACAGCAGAGCATCGTGGTCGAG |
| *Rv0623* | GCTGAGTATCAAGCACCCGGAA | TAACCACTGCCTCGGTCAACGTCT |
| *Rv0909* | AGGTAAAGAACCTGCTGTCGCAGA | GGCGTCAGAATAATTGCCTTGCGT |
| *Rv1103c* | CCAAACTGGTACCATCTGTGAAGTG | TCGTCGTCGATGAACGCGACAAT |
| *Rv1113* | GTTGACGACGCCTTGTTAGCCAAA | ACCCGGACCAGTGTCTGCAA |
| *Rv2009* | TTGTGTCACGGACCAACATCGAGA | CTCATCGTTGCTGAAGTCGAAACC |
| *Rv1241* | GATGAAGCAGGTCATCAACGATGC | AGTTCGTCGGCCAACTTGTTGAAG |
| *Rv1247c* | TGACACACGAGCGCATCACGATAA | TAGCACCTCCAGCGTTTCCTCGAT |
| *Rv1560* | TATGTCGCGCACCAACATCGACAT | GAGCAGAAACTCACGGCTCAACG |
| *Rv1943c* | AGACCTCATCAATTCGTCCAGCGA | AGTAGTGCTCAGCCGTATGCGATT |
| *Rv1955* | CATCAAACCGATTCGTGGCGACAT | TGGGAGTCTTCTGCTGGTTCTTGT |
| *Rv1991A* | ATGAGTCGGTCCGAGTTCTTCACGA | TCCATGGTTTCTAGCACGCGGTAT |
| *Rv2103c* | TATCCTGGCGAGAATGCTGACGTA | AATCGGCCGAAGTCACTGTCGTA |
| *Rv2530A* | CGCACCACGTTGCAGATTGATGAT | AATCCGTCAACCTCGACAATCCCA |
| *Rv2547* | AACTCATCCGACGCGCAATTCA | AATGGCGTCGACATACTCGGT |
| *Rv2653c* | GATGGTGGCGACATCACACGAAAT | TCACTGTTTGCTGTCGGGTTCGT |
| *Rv2758c* | TAGTAGTATGCTCTCCGGGTGTGA | GGCATGCACGGTGTCTTTCTTTGT |
| *Rv2829c* | TCGACTCGCATGTGGCCTACT | AAGCCAGCTCGAACCACGAAAT |
| *Rv2865* | ACAGGACCAGATCACCATCACCAA | AGTACAGCGTCTCCTGCAACGATT |
| *Rv2872* | AGTTAGCGTCCGATGTTGATGCGA | AACCAGGTTGCGTTGTTCTCTAGC |
| *Rv3407* | AGGAACTTGGCGTCACCAACAAAG | TTCGTTGAGAACATCGGACAGGGT |
