# Supplementary material for: Comprehensive Functional Analysis of Mycobacterium tuberculosis Toxin-Antitoxin Systems: Implications for Pathogenesis, Stress Responses, and Evolution
Source: PLoS Genet. 2009 Dec 11;5(12):e1000767. doi: 10.1371/journal.pgen.1000767 (PMC2781298; doi:10.1371/journal.pgen.1000767)
Supplement: Text S1 — Supporting methods. (0.04 MB DOC) [file pgen.1000767.s010.doc]

**Supporting Methods**

**Strains and plasmids.** *M. tuberculosis* (Erdman) and *M. smegmatis* (mc^2^155) [1] were cultured as previously described. Inducible expression in *M. smegmatis* was done using the acetamidase promoter and the tetracycline-inducible promoter [2,3]. *E. coli* BL21(DE3) pLysS cells were used for protein expression (Invitrogen) and *E. coli* DH5α cells were used for cloning.

**Genome sequences.** The fully sequenced and annotated genomes of *Mycobacterium bovis AF2122/97* (NC_002945*)*, *Mycobacterium avium* 104 (NC_008595), *Mycobacterium avium paratuberculosis* str. k10 (NC_002944), *Mycobacterium smegmatis* (NC_008596), *Mycobacterium vanbaalenii* (NC_008726), *Mycobacterium gilvum* (NC_009338), *Mycobacterium abscessus* ATCC 19977T (NC_010397), *Mycobacterium sp*. MCS (NC_008146), *Mycobacterium sp.* JLS (NC_009077), *Mycobacterium sp. KMS* (NC_008705), *Mycobacterium ulcerans* Agy99 (NC_008611), *Mycobacterium marinum* M (NC_010612), *Mycobacterium leprae* TN (NC_002677), and *Mycobacterium tuberculosis* H37Rv (NC_000962) were downloaded from the NCBI database. The latest assemblies of the unfinished genomes for *Mycobacterium canetti* strain CIPT140010059, *Mycobacterium africanum* strain GM041182, and *Mycobacterium microti* strain OV254 were downloaded from the Sanger Institute website (http://www.sanger.ac.uk/) with permission from Dr. Julian Parkhill, Director of Sequencing. The current assembly of the *Mycobacterium kansasii* ATCC 12478 genome (CM000636.1) was downloaded from the NCBI database and used with the permission of Dr. Marcel Behr.

**Reciprocal BLAST searches with toxin query sequences**. We used standard BLASTP to search protein databases of the above genomes for homologs of the *M. tuberculosis* toxin proteins identified in this study. For the finished genomes, we first searched the previously annotated protein databases for each genome. Because the toxin genes are small and potentially missed in these annotations, a second protein database of open reading frames > 80 amino acids was generated for each genome using the open reading frame (ORF) identifying algorithm in Artemis [4]. Two previously un-annotated toxins in the *M. bovis* genome were identified in this manner. For the unfinished genomes, a protein database was generated using the ORF identifying algorithm in Artemis [4], again identifying all ORFs > 80 amino acids. To define potential orthologs, BLAST hits identified in the first pass were used to query the *M. tuberculosis* protein database and BLAST best reciprocal hits were retained as potential orthologs. A cut-off expectation (E)-value of 10^-6^  was used in this analysis. Because of the numerous pseudogenes encoded in the *M. leprae* genome, we did not use BLASTP to identify potential toxin homologs. Rather, we used a precomputed whole genome TBLASTX available from the Sanger Institute and identified toxin homologs and orthologs based on BLAST homology and genomic context analyses (see below).

**Genomic context analysis of homologous TA systems.** To further differentiate orthologs from other homologs, we determined whether the genomic context of top reciprocal BLAST hits was conserved. For the completed genomes present in the MicrobesOnline database [5], we used the Genome and Tree Browser functions to analyze the genomic context of each BLAST hit. For the unfinished genomes, TBLASTX comparisons between each genome and the *M. tuberculosis* genome were generated using the big_blast.pl script (http://www.sanger.ac.uk/Software/ACT/manual/ start.html) and an E-value of 10^-6^. Blast hits were then visualized within their genomic context using the Artemis Comparison Tool or ACT [6]. For *M. leprae*, we used a precomputed whole genome TBLASTX comparison between the *M. tuberculosis* H37Rv and *M. leprae* genomes available on the Sanger Institute website (http:// www.sanger.ac.uk/Projects/M_leprae/comparison.shtml) and visualized the BLAST hits using ACT [6]. This comparison file was generated by a complete genome vs. genome TBLASTX comparison, and thus represents CDS-independent protein-protein similarities between the two genomes. For the purposes of this study, orthologs were defined as best reciprocal BLAST hits that share similar genomic context while homologs were defined as best reciprocal BLAST hits that do not share similar genomic locations.

**Phylogenetic analysis of mycobacterial rRNA sequences.** Sequences encoding the 16S rDNA were retrieved from public databases and aligned using the Clustal X software package [7]. Phylogenetic trees were constructed using the Neighbor Joining inference method and bootstrap analysis (1000 replicates) was performed using the Phylip software package [8]. Resulting trees were drawn using the Interactive Tree of Life web interface (http://itol.embl.de/).

**Supporting References**

1. Snapper SB, Melton RE, Mustafa S, Kieser T, Jacobs WR, Jr. (1990) Isolation and characterization of efficient plasmid transformation mutants of *Mycobacterium smegmatis*. Mol Microbiol 4: 1911-1919.

2. Ehrt S, Guo XV, Hickey CM, Ryou M, Monteleone M, *et al.* (2005) Controlling gene expression in mycobacteria with anhydrotetracycline and Tet repressor. Nucleic Acids Res 33: e21.

3. Daugelat S, Kowall J, Mattow J, Bumann D, Winter R, *et al.* (2003) The RD1 proteins of *Mycobacterium tuberculosis*: expression in *Mycobacterium smegmatis* and biochemical characterization. Microbes Infect 5: 1082-1095.

4. Rutherford K, Parkhill J, Crook J, Horsnell T, Rice P, *et al.* (2000) Artemis: sequence visualization and annotation. Bioinformatics 16: 944-945.

5. Alm EJ, Huang KH, Price MN, Koche RP, Keller K, *et al.* (2005) The MicrobesOnline Web site for comparative genomics. Genome Res 15: 1015-1022.

6. Carver TJ, Rutherford KM, Berriman M, Rajandream MA, Barrell BG, *et al.* (2005) ACT: the Artemis Comparison Tool. Bioinformatics 21: 3422-3423.

7. Chenna R, Sugawara H, Koike T, Lopez R, Gibson TJ, *et al.* (2003) Multiple sequence alignment with the Clustal series of programs. Nucleic Acids Res 31: 3497-3500.

8. Felsenstein J (2005) Using the quantitative genetic threshold model for inferences between and within species. Philos Trans R Soc Lond B Biol Sci 360: 1427-1434.

9. Makarova KS, Wolf YI, Koonin EV (2009) Comprehensive comparative-genomic analysis of Type 2 toxin-antitoxin systems and related mobile stress response systems in prokaryotes. Biol Direct 4: 19.

10. Sevin EW, Barloy-Hubler F (2007) RASTA-Bacteria: a web-based tool for identifying toxin-antitoxin loci in prokaryotes. Genome Biol 8: R155.

11. Tsolaki AG, Hirsh AE, DeRiemer K, Encisco JA, Wong MZ, *et al.* (2004) Functional

and evolutionary genomics of *Mycobacterium tuberculosis*: insights from genomic deletions in 100 strains. Proc Natl Acad Sci U S A 101: 4865-4870.
